# Synthesis of Bispecific Conjugates by ADP‐Ribosyl Cyclases

**DOI:** 10.1002/advs.202518329

**Published:** 2025-12-21

**Authors:** Sunny H. Kim, Arshad J. Ansari, Lei Zhang, Guoyun Kao, Thuc Oanh Hoang, Zeyu Zhang, Srinivasarao Singireddi, Benjamin B. Katz, Yong Zhang

**Affiliations:** ^1^ Department of Pharmacology and Pharmaceutical Sciences Alfred E. Mann School of Pharmacy and Pharmaceutical Sciences University of Southern California Los Angeles CA USA; ^2^ Department of Chemistry University of California Irvine CA USA; ^3^ Department of Chemistry Dornsife College of Letters Arts and Sciences University of Southern California Los Angeles CA USA; ^4^ Norris Comprehensive Cancer Center University of Southern California Los Angeles CA USA; ^5^ Research Center for Liver Diseases University of Southern California Los Angeles CA USA

**Keywords:** Bispecific conjugate, CD38, immunotherapy, prostate cancer

## Abstract

Armed with two distinct targeting moieties, artificially created bispecific agents can uniquely modulate various processes and events implicated in human health and diseases. Despite recent approvals of multiple bispecific therapeutics, generation of homogeneous dual‐targeting constructs with desired pharmacological properties remains technically challenging. Here, we report a strategy for synthesis of bispecific agents by utilizing CD38, a member of the ADP‐ribosyl cyclase family, and its covalent inhibitor. A model ADP‐ribosyl cyclase‐enabled bispecific conjugate (ARC‐BsC) against human T‐cell CD3 and prostate‐specific membrane antigen (PSMA) is generated through site‐specific conjugation of an anti‐CD3 antibody‐CD38 fusion with the CD38 covalent inhibitor derivatized by a PSMA small‐molecule ligand. The resulting ARC‐BsC can redirect and activate cytotoxic T cells toward killing PSMA‐expressing tumor cells, eliciting highly potent and selective anti‐cancer immunity in vitro and in vivo. This proof‐of‐concept work demonstrates ARC‐BsC as a potentially general approach for the development of bispecific therapeutics with diverse applications.

## Graphical abstract

Genetic fusion of a CD38 enzymatic domain to antibody scaffolds allows site‐specific conjugation of small‐molecule ligands. Using this approach, a PSMA ligand DUPA is conjugated with an anti‐human CD3 Fab antibody. The resulting bispecific anti‐CD3‐DUPA conjugate facilitates recruitment and activation of cytotoxic T cells against PSMA‐expressing prostate tumors for potent elimination with high specificity.

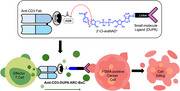

## 1 Introduction

Chemically or genetically linking a monoclonal antibody with a targeting group such as small‐molecule ligand, aptamer, peptide, or another antibody creates an artificial bispecific agent [1, 2, 3, 4, 5, 6, 7]. By engaging with two distinct molecular targets on the same or different types of cells, bispecific agents promote simultaneous modulation of two signaling pathways, internalization of surface proteins for degradation, protein‐protein interactions, biological barrier crossing, and cell recruitment [8, 9, 10, 11, 12, 13, 14, 15]. As a result of their unique modes of action, these synthetic conjugates or genetic fusions with dual‐targeting capabilities find broad applications in human health and attract considerable research interest [16, 17, 18, 19, 20, 21, 22, 23].

Despite recent successes in the clinic, development of bispecific therapeutics remains challenging [16, 24, 25]. Genetically fused bispecific antibodies tend to suffer from low stability and suboptimal activity, owing to incompatible physicochemical properties between two antibody scaffolds and limited control of orientation and geometry of fusion proteins [15, 26, 27, 28, 29, 30, 31]. While chemical conjugation may address issues arising from genetic fusions, synthetic approaches could involve difficult procedures and generate heterogeneous products due to complex and inefficient chemistries [32, 33, 34, 35, 36, 37, 38]. Therefore, new technologies for producing homogeneous bispecific therapeutics with desired characteristics are highly demanded.

As a type II transmembrane protein, CD38 is a member of the ADP‐ribosyl cyclase family [39, 40, 41]. Its extracellular domain catalyzes rapid synthesis of cyclic ADP‐ribose (cADPR) and ADPR from nicotinamide adenine dinucleotide (NAD^+^) [42, 43, 44, 45, 46]. An analogue of NAD^+^ featuring a 2′‐Cl D‐arabinose in place of the D‐ribose of nicotinamide riboside moiety (2′‐Cl‐araNAD^+^) was recently synthesized as a potent covalent inhibitor of CD38 [47]. Following dissociation of the nicotinamide leaving group, the resulting ADP‐2′‐Cl‐arabinose forms a stable ester bond with the side chain of glutamic acid 226 (E226) residue at the CD38 active site [47]. The tubulin inhibitor auristatin covalently attached to adenine N6 of 2′‐Cl‐araNAD^+^ could be conjugated at the E226 site of CD38 catalytic domains genetically fused to antibodies for intracellular delivery [47, 48]. We thus envisioned that 2′‐Cl‐araNAD^+^ functionalized with a targeting molecule may facilitate generation of bispecific conjugates by harnessing CD38 catalysis. To test this hypothesis, 2‐[3‐(1,3‐dicarboxypropyl)ureido]pentanedioic acid (DUPA) with nanomolar affinity for prostate‐specific membrane antigen (PSMA) as a model ligand along with an anti‐human CD3 model antibody (clone: UCHT1) were chosen [2, 49]. By utilizing the generated anti‐CD3 antibody‐CD38 fusion and 2′‐Cl‐araNAD^+^‐DUPA conjugate, an ADP‐ribosyl cyclase‐enabled bispecific conjugate (ARC‐BsC) was facilely synthesized. The resulting anti‐CD3‐DUPA ARC‐BsC displays selective and potent anti‐tumor immunity through redirecting and activating cytotoxic T cells against PSMA‐expressing prostate cancer (PCa) cells, representing a new strategy for developing bispecific therapeutics.

## 2 Results

**FIGURE 1:**
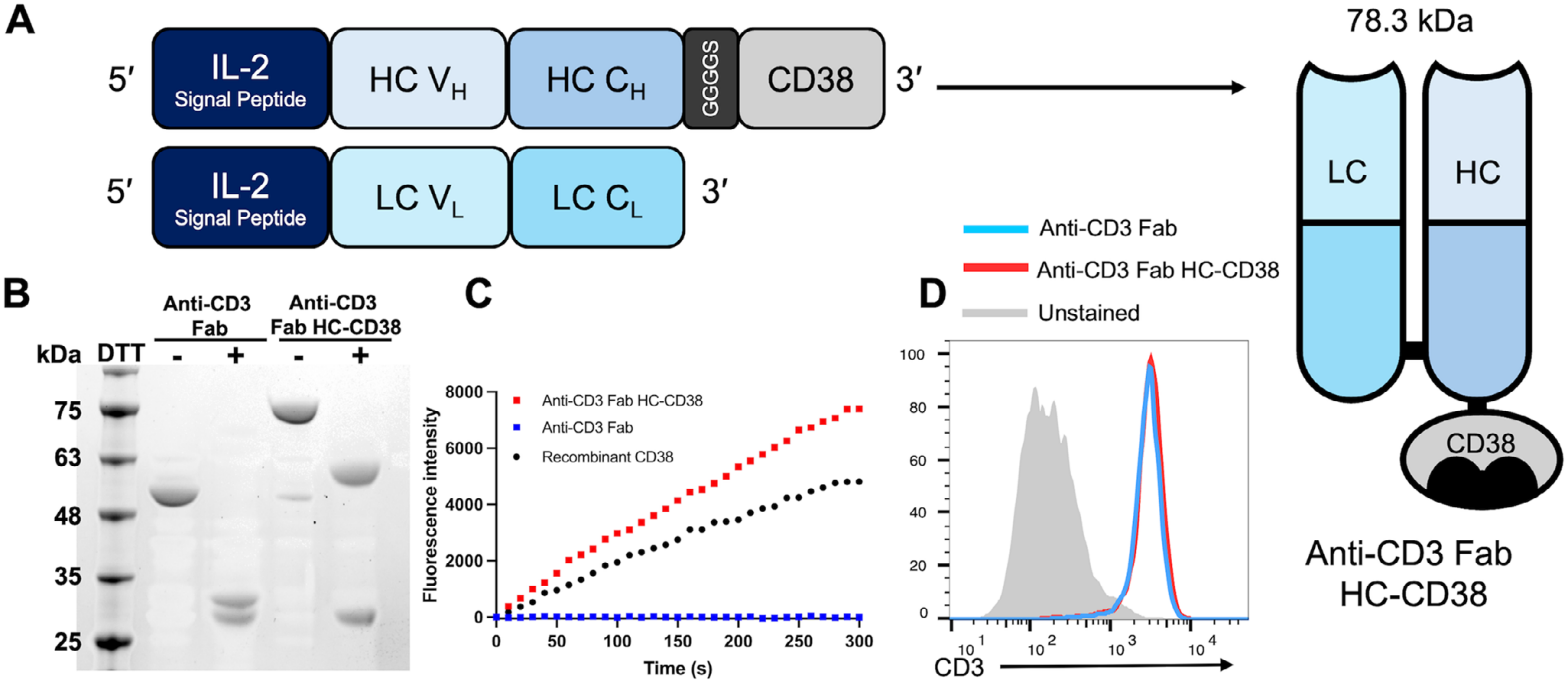
Generation of anti‐CD3 Fab HC‐CD38 fusion. (A) Schematic diagram of anti‐CD3 Fab HC‐CD38 fusion design. (B) Coomassie blue‐stained SDS‐PAGE gel image of purified anti‐CD3 Fab antibodies without and with HC‐CD38 fusion. (C) CD38 enzymatic activity of anti‐CD3 Fab HC‐CD38 measured by NGD^+^‐based fluorescence assays. Anti‐CD3 Fab HC‐CD38 (50 nM) was added into NGD^+^ (100 µM) in PBS, followed by measurements of fluorescence intensity at 410 nm. Anti‐CD3 Fab and recombinant CD38 extracellular domain were included as controls. (D) Flow cytometry of the binding to CD3^+^ Jurkat cells by anti‐CD3 Fab antibodies without and with HC‐CD38 fusion.

Bispecific T‐cell engagers that simultaneously interact with T‐cell marker CD3 and tumor‐associated antigens can induce notable cancer‐specific cellular immunity and have emerged as an appealing class of cancer immunotherapy [15, 50, 51, 52]. Considering their great potential for clinical translation, we undertook the design and generation of a model ARC‐BsC recognizing T‐cell CD3 as well as PSMA overexpressed on most PCa tumors [53, 54, 55]. Currently, few efficacious treatments are available for patients with unresectable and metastatic PCa [56, 57]. To create an anti‐CD3 antibody‐CD38 fusion protein, an anti‐CD3 UCHT1 fragment antigen‐binding (Fab) antibody was selected over the full‐length immunoglobulin G (IgG) and single‐chain fragment variable (scFv) formats in balancing serum stability and tissue penetration capability. The extracellular domain of human CD38 was then genetically fused to C‐terminus of the anti‐CD3 Fab heavy chain (HC) in order to minimize effects of the CD38 fusion on CD3 antigen binding and pairing of antibody HC and light chain (LC) (Figure 1A). A flexible GGGGS linker was inserted between HC and CD38.

The designed anti‐CD3 Fab HC‐CD38 fusion and anti‐CD3 Fab antibody without CD38 were expressed in Expi293F cells through transiently co‐transfecting HC and LC vectors for respective constructs. Antibodies secreted into culture media were purified by protein G affinity chromatography with a yield of 8 mg L^−1^ for each design. SDS‐PAGE gel analysis revealed comparable sizes of LCs for both constructs and an upward ∼30 kDa shift for the HC of anti‐CD3 Fab HC‐CD38 fusion relative to the HC of anti‐CD3 Fab, consistent with molecular designs (Figure 1B). Enzymatic activity of the fused CD38 was examined using nicotinamide guanine dinucleotide (NGD^+^) as a substrate, which could be converted into fluorescent cyclic GDP‐ribose (cGDPR) by CD38 [58, 59]. NGD^+^‐based fluorescence assays indicated that like the recombinant CD38 extracellular domain, anti‐CD3 Fab HC‐CD38 fusion protein is characterized by robust CD38 catalytic activity (Figure 1C). In contrast, anti‐CD3 Fab without CD38 fusion shows no enzymatic activity with NGD^+^. Flow cytometry indicated comparable binding of anti‐CD3 Fab and anti‐CD3 Fab HC‐CD38 for CD3‐expressing Jurkat cells (Figure 1D). Gel filtration chromatographic analysis reveal little or no aggregation for both constructs (Figure S1). These results support stable expression of bifunctional anti‐CD3 Fab HC‐CD38.

**FIGURE 2:**
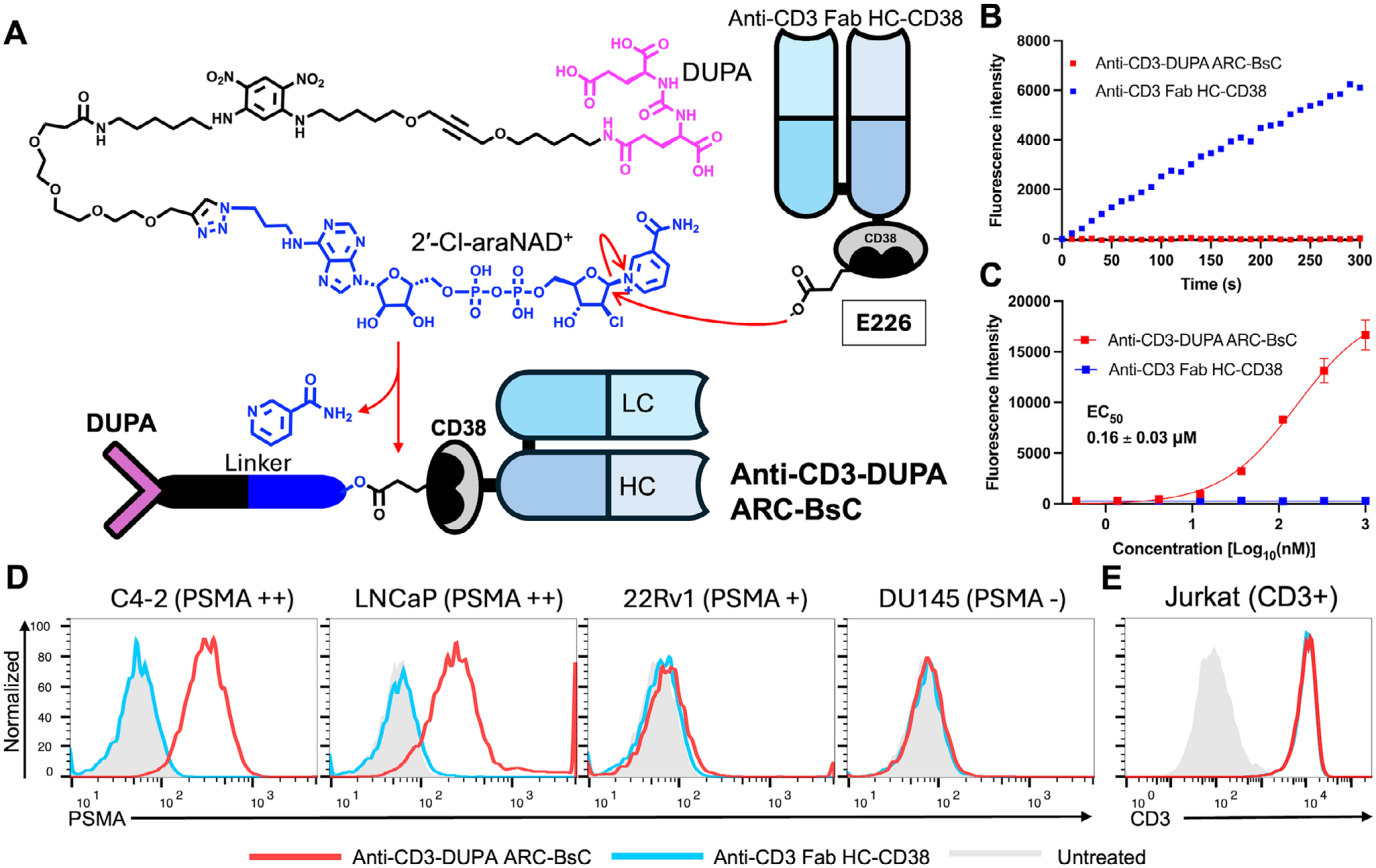
Synthesis and characterization of anti‐CD3‐DUPA ARC‐BsC. (A) Schematic diagram of production of anti‐CD3‐DUPA ARC‐BsC. (B) Residual CD38 catalytic activity upon completion of anti‐CD3‐DUPA ARC‐BsC synthesis measured by NGD^+^‐based fluorescence assays. At the end of conjugation, reaction mixtures containing 50 nM anti‐CD3‐DUPA ARC‐BsC were incubated with NGD^+^ (100 µM) for fluorescence measurements at 410 nm. Anti‐CD3 Fab HC‐CD38 was included as controls. (C) ELISA analysis of the binding of anti‐CD3‐DUPA ARC‐BsC to recombinant PSMA protein. (D) and (E) Flow cytometric analysis of the binding of anti‐CD3‐DUPA ARC‐BsC to PCa cells with varied PSMA express levels (D) and CD3‐positive Jurkat cells (E). Anti‐CD3 Fab HC‐CD38 was included in ELISA and flow cytometry for comparison.

Next, a 2′‐Cl‐araNAD^+^‐DUPA conjugate (∼48 Å) was designed and synthesized. An extended linker composed of ethylene glycol and aliphatic segments was placed between DUPA ligand and 2′‐Cl‐araNAD^+^ to reduce effects of the attached DUPA on 2′‐Cl‐araNAD^+^‐mediated covalent inhibition of the fused CD38 and ensure accessibility of the conjugated DUPA by cell‐surface PSMA as well as efficient binding of DUPA within the deep active site of PSMA (∼20 Å) (Figure 2A). To enhance the binding of DUPA to PSMA, the linker was modified with a 1,3‐dinitrophenyl (DNP) aromatic ring and an alkyne group. The DNP moiety is expected to promote potential *π–π* stacking and electrostatic interactions with residues near the entrance of the DUPA ligand‐binding pocket of PSMA [2, 60]. The centrally positioned alkyne could introduce linker rigidity that may support a favorable orientation of DUPA toward PSMA. Notably, the synthesized 2′‐Cl‐araNAD^+^‐DUPA conjugate displayed increased potency for inhibiting PSMA catalytic activity in comparison with the free DUPA molecule (Figure S2).

The synthesized 2′‐Cl‐araNAD^+^‐DUPA conjugate was then incubated with purified anti‐CD3 Fab HC‐CD38 fusion protein at a molar ratio of 25:1 for producing the anti‐CD3‐DUPA ARC‐BsC (Figure 2A). Conjugation of DUPA with anti‐CD3 Fab HC‐CD38 fusion was completed after 4‐h incubation at room temperature according to CD38 residual activity measured by NGD^+^‐based fluorescence assays (Figure 2B). Mass spectrometric analysis showed an increase for the HC of purified anti‐CD3‐DUPA ARC‐BsC in comparison with that of unconjugated anti‐CD3 Fab HC‐CD38, matching the covalent addition of DUPA ligand via 2′‐Cl‐araNAD^+^ (Figure S3). No mass shifts were observed between the LC of anti‐CD3 Fab HC‐CD38 and anti‐CD3‐DUPA ARC‐BsC (Figure S3).

Binding specificity of anti‐CD3 Fab HC‐CD38 was then evaluated. Compared with unconjugated anti‐CD3 Fab HC‐CD38 displaying no binding to recombinant PSMA protein, anti‐CD3‐DUPA ARC‐BsC can tightly bind to PSMA (EC_50_: 0.16 ± 0.03 µM) based on ELISA assays (Figure 2C). The conjugated DUPA ligand is characterized by a half‐life of more than 12 h in serum (Figure S4). Flow cytometry revealed selective binding to PSMA‐positive PCa cell lines by anti‐CD3‐DUPA ARC‐BsC, which correlates with surface expression levels of PSMA (Figure 2D; Figure S5). By contrast, unconjugated anti‐CD3 Fab HC‐CD38 lacks binding to both PSMA‐positive and ‐negative PCa cells under the same conditions (Figure 2D). Moreover, like anti‐CD3 Fab HC‐CD38 fusion, anti‐CD3‐DUPA ARC‐BsC can specifically bind to CD3‐expressing Jurkat cells (Figure 2E). These results indicated successful generation of anti‐CD3‐DUPA ARC‐BsC dually targeting CD3 and PSMA antigens.

**FIGURE 3:**
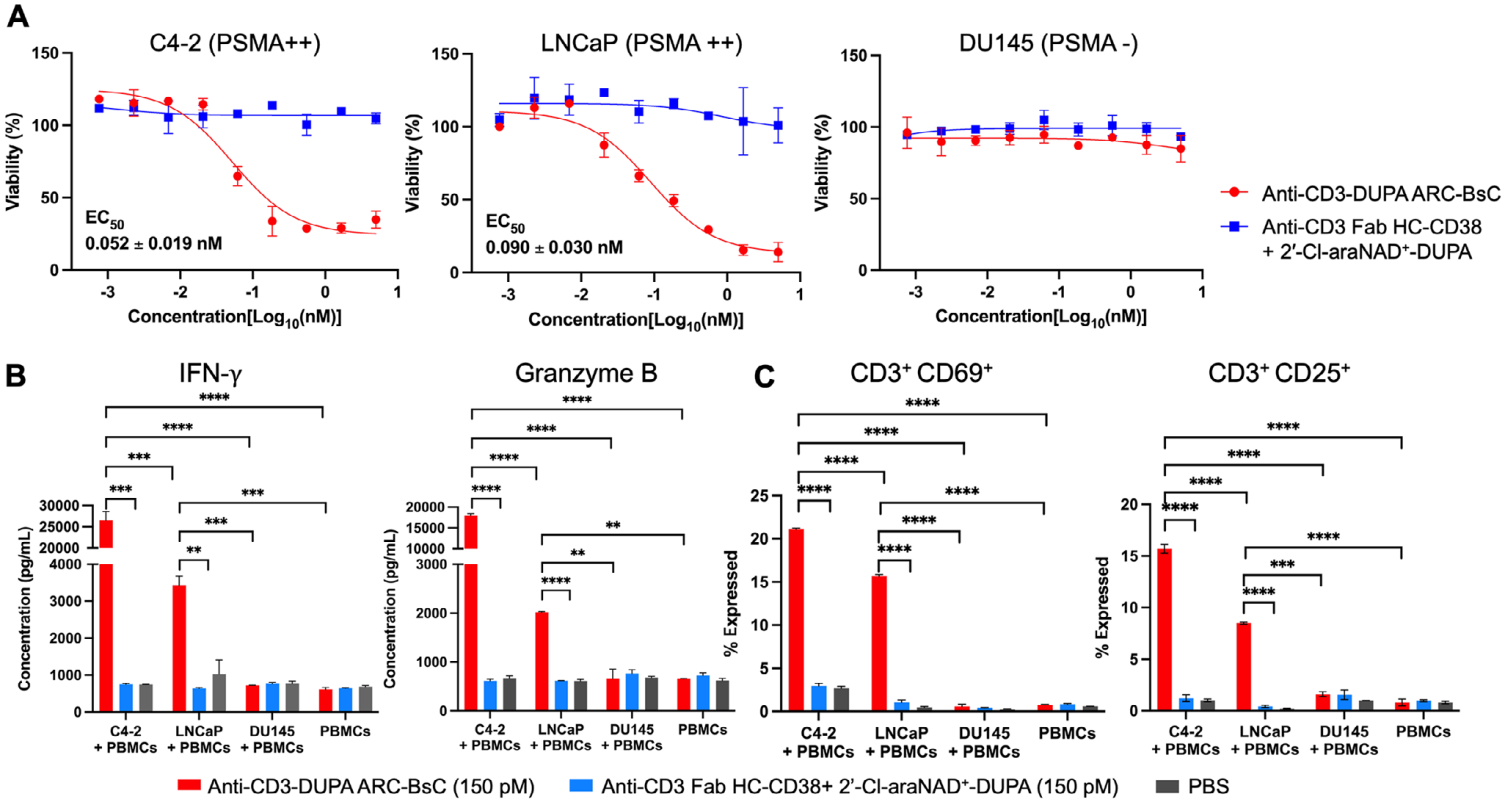
In vitro biological activities of anti‐CD3‐DUPA ARC‐BsC. (A) In vitro cytotoxicity of anti‐CD3‐DUPA ARC‐BsC for PCa cells with varied levels of PSMA expression in the presence of human PBMCs. PCa cells mixed with primary human PBMCs (ratio 1:10) were incubated with anti‐CD3‐DUPA ARC‐BsC at various concentrations for 48 h. After removals of PBMC suspensions, viabilities of PCa cells were measured. A mixture of anti‐CD3 Fab HC‐CD38 and 2ʹ‐Cl‐araNAD^+^‐DUPA conjugate (molar ratio 1:1) was included as controls. (B) and (C) In vitro T‐cell activation by anti‐CD3‐DUPA ARC‐BsC analyzed through secretion levels of IFN‐γ and granzyme B (B) and expression levels of CD69 and CD25 (C). Human PBMCs without and with PCa cells (ratio 10:1) were incubated with 150 pM anti‐CD3‐DUPA ARC‐BsC for 48 h, followed by quantifying IFN‐γ and granzyme B release via ELISA and CD69 and CD25 expression via flow cytometry. PBS and a mixture of anti‐CD3 Fab HC‐CD38 and 2ʹ‐Cl‐araNAD^+^‐DUPA conjugate (molar ratio 1:1) were included as controls. ^**^ *p* < 0.01; ^***^ *p* < 0.001; ^****^ *p* < 0.0001.

In vitro cytotoxicity of anti‐CD3‐DUPA ARC‐BsC was next studied using primary human peripheral blood mononuclear cells (PBMCs) co‐cultured with PCa cell lines with varied levels of PSMA expression. In the presence of human PBMCs, anti‐CD3‐DUPA ARC‐BsC exhibited remarkable potency in killing C4‐2 and LNCaP cells with high levels of PSMA expression (EC_50_: 0.052 ± 0.019 nM for C4‐2 and 0.090 ± 0.030 nM for LNCaP) and reduced cytotoxicity for 22Rv1 cells featuring moderate PSMA expression levels (EC_50_: 0.24 ± 0.12 nM), but spared PSMA‐negative DU145 cells at concentrations up to 5 nM (Figure 3A; Figure S6). As controls, a combination of anti‐CD3 Fab HC‐CD38 and 2′‐Cl‐araNAD^+^‐DUPA (molar ratio 1:1) revealed little or no cytotoxicity against these PCa cell lines under the same conditions. Furthermore, no cytotoxicity was observed for anti‐CD3‐DUPA ARC‐BsC in co‐cultures of PBMCs and non‐PCa cells including MDA‐MB‐468 and HEK293T cells under the same conditions (Figure S7A,B). Additionally, anti‐CD3‐DUPA ARC‐BsC at 5 nM lacked cytotoxic effects on PCa cells with different expression levels of PSMA in the absence of human PBMCs (Figure S7C).

T‐cell activation by anti‐CD3‐DUPA ARC‐BsC was analyzed in the absence and presence of PCa cells by quantifying levels of secreted interferon‐gamma (IFN‐γ) and granzyme B as well as expressed activation marker CD69 and CD25. Quantitative ELISA and flow cytometry revealed that additions of anti‐CD3‐DUPA ARC‐BsC result in substantial secretion of IFN‐γ and granzyme B and significantly elevated CD69^+^ and CD25^+^ T‐cell subpopulations for PBMCs co‐cultured with PSMA‐positive PCa cells (C4‐2 and LNCaP), but not for PBMCs alone or with PSMA‐negative DU145 cells (Figure 3B,C). No significant changes in IFN‐γ and granzyme B release or CD69 and CD25 expression on T cells were seen for cell groups treated by the mixture of anti‐CD3 Fab HC‐CD38 and 2′‐Cl‐araNAD^+^‐DUPA. In addition, anti‐CD3‐DUPA ARC‐BsC induced modest T‐cell activation for PBMCs co‐cultured with 22Rv1 PCa cells expressing lower levels of PSMA according to secreted IFN‐γ and granzyme B and CD69 and CD25 expression (Figure S8). These data support superb in vitro activity and specificity of anti‐CD3‐DUPA ARC‐BsC in eliciting T‐cell immunity toward PSMA‐positive cancer cells.

**FIGURE 4:**
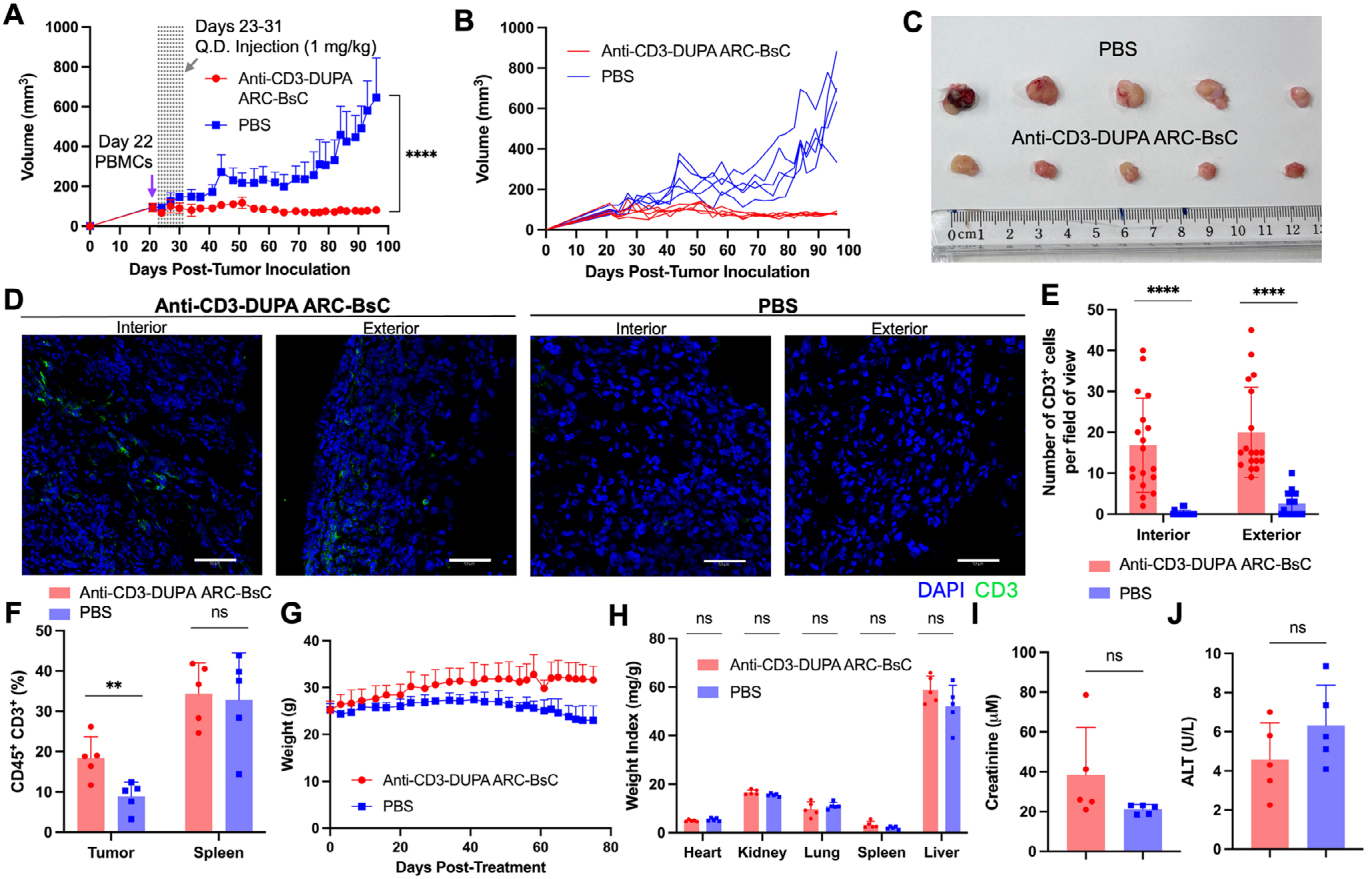
In vivo efficacy and toxicity of anti‐CD3‐DUPA ARC‐BsC in mice. (A) Tumor growth curves after treatment measured by a caliper. Male NSG (n = 5) mice implanted with C4‐2 cell‐derived tumors in right flanks were grafted with human PBMCs on day 22, followed by daily injections (i.v.) of anti‐CD3‐DUPA ARC‐BsC (1 mg kg^−1^) or PBS on day 23–31. (B) Tumor growth curves for individual animals following treatment. (C) Photograph of tumors collected from mice in each group at the end of the study. (D) Representative immunofluorescence images of the interior and exterior portion of tumor cryosections from each group. Scale bars, 50 µm. (E) Quantitative representations of numbers of CD3^+^ cells from each field of view in the interior and exterior portion of the tumor cryosections (6 fields of view per region and three mice per group). (F) T‐cell infiltration in tumor and spleen samples from each group analyzed by flow cytometry. (G) Mice body weight monitored after treatment. (H) Weight indices calculated for major organs collected at the end of the study. (I) and (J) Plasma levels of creatinine (I) and ALT (J) at the end of the study. ns, not significant, *p* > 0.05; ^**^ *p* < 0.01; ^****^ *p* < 0.0001.

In vivo efficacy and toxicity of anti‐CD3‐DUPA ARC‐BsC were evaluated in a mouse xenograft model. Male mice implanted with human C4‐2 cell‐derived tumors were grafted with human PBMCs when tumor sizes measured with a caliper reached ∼100 mm^3^, followed by daily treatment with phosphate‐buffered saline (PBS) or anti‐CD3‐DUPA ARC‐BsC (1 mg kg^−1^) for nine days. Subsequent monitoring of tumor sizes indicated that relative to PBS‐treated animals with continuously expanding tumors, mice administered with anti‐CD3‐DUPA ARC‐BsC presented significant inhibition of tumor growth (Figure 4A–C). Immunofluorescence imaging revealed tumor‐infiltrating T cells from anti‐CD3‐DUPA ARC‐BsC‐treated mice, but few or no infiltrating T cells in tumors from mice receiving PBS vehicle (Figure 4D,E). Similarly, flow cytometry supported considerably higher levels of T‐cell infiltration for tumors from the group treated with anti‐CD3‐DUPA ARC‐BsC than those for tumors of PBS‐treated mice (Figure 4F). Furthermore, unlike PBS‐treated group with declining body weight, mice receiving anti‐CD3‐DUPA ARC‐BsC gained weight over the course of study (Figure 4G). At the end of the study, major organ weight indices and levels of creatinine (kidney damage marker) and alanine aminotransferase (ALT) (liver damage marker) in plasma were comparable between PBS and anti‐CD3‐DUPA ARC‐BsC treatment groups (Figures 4H–J). Taken together, these results demonstrate excellent in vivo anti‐tumor potency and adequate safety profiles for anti‐CD3‐DUPA ARC‐BsC.

## 3 Discussion

This study presents a facile approach to the generation of bispecific agents by exploiting CD38 catalytic domain and its covalent inhibitor 2′‐Cl‐araNAD^+^. A model bispecific agent for potential PCa immunotherapy was created through combining anti‐CD3 Fab HC‐CD38 fusion protein with 2′‐Cl‐araNAD^+^‐DUPA conjugate. The anti‐CD3‐DUPA ARC‐BsC could be efficiently synthesized within 4 h at room temperature via a single‐step site‐specific conjugation. CD38 genetic fusion coupled with the 2′‐Cl‐araNAD^+^ derivative establishes a feasible strategy for rapid and efficient development of bispecific therapeutics. Compared with other established genetic and chemical methods, ARC‐BsC represents a modular platform for generating homogeneous conjugates through one‐step enzymatic reactions under mild conditions, which needs simple incorporation of CD38 extracellular domain and synthesis of NAD^+^ derivatives. Unlike other genetically fused tags derived from non‐human proteins or highly engineered human enzymes, ARC‐BsC exploits an aglycosylated human CD38, reducing potential immunogenicity. In contrast to those enzyme‐mediated conjugation technologies requiring removals of added enzymes after reactions, ARC‐BsC eliminates the purification step and simplifies the production process. While CD38 fusion facilitates site‐specific conjugation, the fused CD38 domain could potentially destabilize antibody scaffolds and reduce tissue penetration due to increased sizes.

To enable 2′‐Cl‐araNAD^+^‐mediated conjugation, CD38 needs to be fused with antibody scaffolds. Compared with anti‐CD3 Fab, fusion of a CD38 catalytic domain to the C‐terminus of anti‐CD3 Fab HC causes no changes to the expression yield. Also, the presence of Fab domain at CD38 N‐terminus leads to no reduction in its enzymatic activity in comparison with recombinant CD38 catalytic domain. These results suggest that the fusion of anti‐CD3 Fab with CD38 has minimal effects on antibody stability and CD38 catalytic activity. In addition, unconjugated anti‐CD3 Fab HC‐CD38 and anti‐CD3‐DUPA ARC‐BsC showed comparable binding to CD3^+^ Jurkat cells, implicating little impact on CD3 binding from the conjugated DUPA ligand.

The generated anti‐CD3‐DUPA ARC‐BsC is characterized by picomolar potency in activating cytotoxic T cells toward attacking PSMA‐positive PCa cells. T‐cell activation triggered by anti‐CD3‐DUPA ARC‐BsC is dependent on PSMA antigen expression on target cells. Through recruiting T cells in circulation to PSMA‐expressing tumors, anti‐CD3‐DUPA ARC‐BsC induces strong cellular immunity and consequently tumor suppression in animals.

CD38 was genetically fused with anti‐CD3 Fab for DUPA conjugation. This Fab antibody‐based ARC‐BsC displays potent in vitro and in vivo biological activities. To increase binding affinity and/or efficacy, DUPA ligands could be attached to anti‐CD3 Fab antibodies carrying additionally fused CD38 domains. Also, DUPA could be conjugated with CD38 fused to anti‐CD3 scFv and IgG antibodies, producing scFv‐ and IgG‐derived ARC‐BsCs. Due to smaller sizes and likely reduced stability, ARC‐BsCs with scFv antibodies may have shorter half‐lives in blood, but improved activities in triggering T‐cell immunity and tissue penetration. ARC‐BsCs based on IgG fusion may show reduced T‐cell activation potencies and tissue penetration because of increased molecular weights. However, their half‐lives could be prolonged, and the Fc domain can afford additional effector functions.

Anti‐CD3‐DUPA ARC‐BsC exhibits great performance in the mouse C4‐2 xenograft model with grafted human PBMCs. But potential on‐target, off‐tumor toxicity of anti‐CD3‐DUPA ARC‐BsC remains unclear due to limitations of the NSG xenograft models and presence of CD38‐interacting proteins on cell surface [61, 62]. Additional controls, dosages, and cellular and animal models would be needed for comprehensive characterization of efficacy, specificity, and safety profiles. Additionally, the ARC‐BsC technology could be extended to various targeting moieties from antibodies to small molecules through CD38 genetic fusion and/or 2′‐Cl‐araNAD^+^ functionalization for creation of bispecific agents with distinct modes of action.

## 4 Conclusion

In conclusion, anti‐CD3‐DUPA ARC‐BsC was readily synthesized by utilizing anti‐CD3‐CD38 fusion protein and DUPA‐functionalized 2′‐Cl‐araNAD^+^. It features potent in vitro and in vivo anti‐cancer activity by eliciting PSMA^+^ cell‐specific T cell immune responses. ARC‐BsC may provide a versatile platform for developing bispecific therapeutics to address unmet medical needs.

## 5 Experimental Methods

### 5.1 Materials

L‐glutamine (25‐005‐CI), RPMI 1640 medium (10‐040‐CV), and Matrigel matrix (354248) were purchased from Corning, NY. Fetal bovine serum (FBS) (A56707‐01), AccuPrime *Pfx* DNA polymerase kits (12344024), Opti‐MEM medium (31985070), bovine serum albumin (BSA) (BP9703‐100), 3‐(4,5‐dimethylthiazol‐2‐yl)‐2,5‐diphenyltetrazolium bromide (MTT) (M6494), QuantaBlue fluorogenic substrate kits (15169), goat anti‐mouse IgG (H+L) cross‐adsorbed secondary antibody, Alexa Fluor 488 (A11001), Hoechst 33342 (H3570), and interleukin‐2 (IL‐2) (200‐02‐100UG) were purchased from Thermo Fisher Scientific, MA. FITC anti‐human CD3 antibody (300440), PE anti‐human CD25 antibody (385608), APC anti‐human CD69 antibody (310910), PerCP/Cyanine5.5 anti‐human CD45 antibody (304028), anti‐CD3 antibody (317326), and anti‐CD28 antibody (302934) were purchased from BioLegend, CA. Goat anti‐human kappa‐HRP (2060‐05) and goat anti‐human kappa‐FITC (2060‐02) were purchased from Southern Biotech, AL. NheI (R3131S) and EcoRI (R3101S) restriction enzymes and T4 DNA ligase (M0202S) were purchased from New England Biolabs, MA. Human IFN‐gamma DuoSet ELISA (DY285B) and human granzyme B DuoSet ELISA (DY2906‐05) were purchased from R&D Systems, MN. ZymoPURE maxiprep kits (11‐555) were purchased from ZymoResearch, CA. PEI MAX transfection grade linear polyethylenimine hydrochloride (25765‐1) was purchased from Polysciences Inc., PA. Centrifugal concentrators (UFC903024) were purchased from EMD Amicon, CA. Protein G resin (L00209) was purchased from GenScript, NJ. BalanCD HEK293 medium was purchased from FUJIFILM Biosciences, CA. Nicotinamide guanine dinucleotide (NGD^+^) sodium salt (N5131) was purchased from MilliporeSigma, MA. Recombinant human prostate‐specific membrane antigen (PSMA) protein with a His_6_ tag (PSA‐H52H3) was purchased from ACRO Biosystems, DE. Ninety‐six‐well high‐binding plates (655077) were purchased from Greiner Bio‐one, AT. Multivette 600 lithium heparin gel LH tubes (15.1675.100) were purchased from Sarstedt AG & Co. KG, DE. Optimum cutting temperature (O.C.T.) (4583) formulation was purchased from Sakura Finetek, JP. Phthaladehyde (A131637), spaglumic acid (A766233), and DUPA (A1215704) were purchased from Ambeed Inc, IL.

### 5.2 Cell Culturing

Expi293F cells (RRID:CVCL_D615) were purchased from Thermo Fisher Scientific and cultured in BalanCD HEK293 medium supplemented with L‐glutamine with shaking at 125 rpm, 37°C, and 5% CO_2_. C4‐2 (RRID:CVCL_4782) and Jurkat (RRID:CVCL_0065) cell lines were purchased from the American Type Culture Collection (Manassas, VA) and cultured in RPMI 1640 medium supplemented with 10% FBS. LNCaP (RRID:CVCL_0395) and DU145 (RRID:CVCL_0105) were gifts from Dr. Jean Shih's laboratory at the University of Southern California and cultured in RPMI 1640 medium containing 10% FBS. Human peripheral blood mononuclear cells (PBMCs) were purchased from Charles River Laboratories (Wilmington, MA) and cultured in RPMI 1640 medium with 10% FBS. All cell lines were free of mycoplasma.

### 5.3 Animal Study

All animal procedures were approved by the University of Southern California Institutional Animal Care and Use Committee. Five‐week‐old male NOD.Cg‐*Prkdc^scid^* *Il2rg^1Wjl^*/SzJ (NSG) mice were purchased from The Jackson Laboratory (Bar Harbor, ME).

### 5.4 Chemical Synthesis

Experimental details for chemical synthesis of 2′‐Cl‐araNAD^+^‐DUPA are provided in the Supporting Information.

### 5.5 Molecular Cloning

A DNA fragment encoding anti‐human CD3 antibody (clone: UCHT1) heavy chain (HC) fragment antigen‐binding (Fab) region (V_H_ and C_H1_) was amplified by polymerase chain reaction (PCR) using a previously generated vector expressing anti‐human CD3 immunoglobulin G (IgG) antibody HC as a template [33]. To generate a DNA fragment encoding anti‐CD3 Fab HC‐CD38 fusion, overlap extension PCR was performed using amplified DNA fragments of anti‐CD3 Fab antibody HC and CD38 extracellular domain [43]. A GGGGS linker was added between anti‐CD3 Fab antibody HC and CD38. Resulting DNA fragments of anti‐CD3 Fab HC and anti‐CD3 Fab HC‐CD38 fusion were ligated in‐frame with pFUSE vectors between NheI and EcoRI restriction sites by T4 ligase. Sequence‐verified plasmids were then prepared using ZymoPURE plasmid maxiprep kits.

### 5.6 Protein Expression and Purification

Expi293F cells were cultured in BalanCD HEK293 medium for 3–5 passages with shaking at 125 rpm, 37°C and 5% CO_2_. To transiently transfect Expi293F cells with antibody plasmids, cell density was adjusted to be 5×10^6^ cells mL^−1^ in 120 mL of culture media. The plasmid (120 µg) encoding anti‐CD3 Fab HC or anti‐CD3 Fab HC‐CD38 fusion together with the plasmid expressing anti‐CD3 light chain (120 µg) generated previously were added to 12 mL of Opti‐MEM medium along with 960 µL of 1.11 g L^−1^ PEI MAX solution [33]. The solution was well mixed, incubated at room temperature for 20 min, and then added to cell culture. After 2‐h incubation with shaking at 125 rpm, 37°C, and 5% CO_2_, additional 120 mL of BalanCD HEK293 medium was added to flasks, yielding a final cell density of 2.5×10^6^ cells mL^−1^ in 240 mL. Transfected cells were cultured for 5 day at 37°C and 5% CO_2_. Culture media were collected on day 5 post transfection. A series of centrifugation (100×g for 10 min and 4000×g for 30 min) were performed to remove cell debris from media. Supernatants were then loaded onto columns packed with 1 mL of protein G resin for antibody purification. Antibodies were eluted with 5 mL of 100 mm glycine (pH 2.7) and neutralized with 0.5 mL of 1 m Tris (pH 8.4). Eluted proteins were then buffer exchanged against PBS. Concentrations of purified antibodies were measured using a Nanodrop 2000C spectrometer.

Recombinant CD38 extracellular domain with a C‐terminal His_6_ tag was expressed in Expi293 cells through transient transfection and purified by nickel‐nitrilotriacetic acid (Ni‐NTA) affinity chromatography as previously described [40, 43].

Anti‐CD3 Fab and anti‐CD3 Fab HC‐CD38 were examined by gel filtration chromatography by injecting 200 µg of each construct onto a Superdex 200 Increase 10/300 GL (Cytiva, MA) column in PBS with a flow rate of 0.5 mL min^−1^. Fractions with antibodies were analyzed by SDS‐PAGE gels.

### 5.7 Enzymatic Activity Assays

CD38 catalytic activity of anti‐CD3 Fab HC‐CD38 was analyzed using NGD^+^‐based fluorescence assays. In black 96‐well plates, 50 nM anti‐CD3 Fab HC‐CD38, anti‐CD3 Fab, or recombinant CD38 extracellular domain was mixed with 100 µM NGD^+^ in PBS. Fluorescent intensity was measured with excitation wavelength at 300 nm and emission wavelength at 410 nM for 5 min using a Synergy H1 plate reader (Bio Tek, VT).

### 5.8 Inhibition Activity of 2′‐Cl‐araNAD^+^‐DUPA

Inhibitory activities of 2′‐Cl‐araNAD^+^‐DUPA and free DUPA against PSMA were measured by fluorescence‐based assays. Briefly, recombinant PSMA was diluted to 0.4 µg mL^−1^ in assay buffer (50 mM HEPES, 0.1 M NaCl, pH 7.5) and N‐acetyl‐aspartyl‐glutamate (NAAG) was diluted to 40 µm in assay buffer. PSMA (100 µL) was mixed with the inhibitor starting at 3 µM with threefold serial dilutions, followed by additions of 100 µL of NAAG solution. After 1‐h incubation at 37°C, reactions were quenched by boiling at 95°C for 5 min. Reaction (50 µL) was transferred to black 96‐well plates, followed by additions of 50 µL of 10 mm ortho‐phthaldialdehyde (OPA) in OPA buffer (0.2 m NaOH, 0.1% beta‐mercaptoethanol (v/v)) and incubation at room temperature for 10 min with gentle shaking. Fluorescence intensity was measured with excitation wavelength of 330 nm and emission wavelength at 450 nm using a Synergy H1 plate reader. The three‐parameter nonlinear regression fitting in GraphPad Prism version 10 (GraphPad software, CA) was used to calculate half‐maximal effective concentration (EC_50_) for each inhibitor. The activity was normalized based on PSMA activity in the absence of inhibitor (100%) or substrate (0%).

### 5.9 Synthesis of Anti‐CD3‐DUPA ARC‐BsC

Purified anti‐CD3 Fab HC‐CD38 fusion protein was mixed with 2′‐Cl‐araNAD^+^‐DUPA at a molar ratio of 1:25 in 50 mM Tris buffer (pH 8.4). The reaction solution was incubated at room temperature for 4 h. NGD^+^‐based CD38 activity assays were used to monitor reaction progress. Upon completion of conjugation, free 2′‐Cl‐araNAD^+^‐DUPA conjugate was removed via buffer exchange using 30 kDa‐molecular weight cutoff amicon centrifugal concentrators.

### 5.10 ELISA Analysis of Binding

Recombinant human PSMA (2.5 µg mL^−1^) was coated on black 96‐well high‐binding plates overnight. The next day, wells were washed with 400 µL of PBS with 0.05% Tween‐20 (PBST) for three times. Then, wells were blocked with 300 µL of 3% BSA for 2 h at room temperature, followed by three washes using PBST. Anti‐CD3‐DUPA ARC BsC or anti‐CD3 Fab HC‐CD38 at different concentrations was added and incubated for 1 h. After another three washes, goat anti‐human kappa‐HRP was added and incubated for 1 h. Lastly, wells were washed four times and added with 100 µL of QuantaBlu fluorogenic substrates. Fluorescence intensity was measured with excitation wavelength of 325 nm and emission wavelength at 420 nm using a Synergy H1 plate reader. The three‐parameter nonlinear regression fitting in GraphPad Prism version 10 (GraphPad software, CA) was used to calculate EC_50_ for each construct.

### 5.11 Serum Stability Analysis

Anti‐CD3‐DUPA ARC‐BsC (30 µm (2.3 mg mL^−1^)) was incubated in 100% FBS at 37°C for up to 96 h. Fractions of the mixture were collected at 0, 6, 12, 24, 48, 72, and 96 h. Each fraction was diluted 100‐fold with 1% BSA and assessed for binding to recombinant PSMA via ELISA assays as described above. Standard curves were generated using anti‐CD3‐DUPA ARC‐BsC at concentrations of 500 nm (40 µg mL^−1^) to 0.7 nm (0.05 µg mL^−1^) (threefold serial dilution). The half‐life was calculated with one‐phase decay nonlinear regression fitting in GraphPad Prism version 10 (GraphPad software, CA).

### 5.12 Flow Cytometric Analysis of Binding

Four different cell lines with varied PSMA expression levels including C4‐2 (PSMA ++), LNCaP (PSMA ++), 22Rv1 (PSMA +), and DU145 (PSMA ‐) were used to evaluate binding of anti‐CD3‐DUPA ARC BsC to PSMA. CD3‐positive Jurkat cells were used to assess binding of anti‐CD3‐DUPA ARC BsC for CD3. Cells (5 × 10^5^) of each cell line were resuspended in 100 µL of 2% FBS in PBS and then incubated with 200 nM of anti‐CD3‐DUPA ARC BsC or anti‐CD3 Fab HC‐CD38 for 30 min on ice. Cells were washed with ice‐cold 2% FBS in PBS, stained with goat anti‐human kappa‐FITC, and then washed three times with 2% FBS in PBS. Stained cells were analyzed using a BD Fortessa X20 cell analyzer (BD Biosciences, CA). Data were processed with FlowJo version 10 software.

### 5.13 Mass Spectrometric Analysis

Anti‐CD3 Fab HC‐CD38 and anti‐CD3‐DUPA ARC‐BsC were analyzed by liquid chromatography‐mass spectrometry by following a previously reported method [47].

### 5.14 In Vitro Cytotoxicity Assays

PSMA‐positive or ‐negative cells (C4‐2, LNCaP, 22Rv1, DU145, MDA‐MB‐468, and HEK293T) (10^4^ cells per well) were mixed with primary human PBMCs (10^5^ cells per well) in 96‐well cell culture plates. Anti‐CD3‐DUPA ARC‐BsC or a combination of anti‐CD3 Fab HC‐CD38 and 2′‐Cl‐araNAD^+^‐DUPA (molar ratio 1:1) at nine different concentrations (threefold serial dilutions beginning at 5 nm) were added to each well with duplicates. PBS‐treated cell mixtures were used as 100% viability controls. Wells with only human PBMCs plus PBS treatment were included as 0% viability controls. After 48‐h incubation at 37°C in a 5% CO_2_ incubator, PBMC suspensions were removed and wells were carefully washed with PBS twice. Following additions of 90 µL of the complete medium and 10 µL of 12 mM of MTT solution, plates were incubated for 3 h at 37°C. Next, 100 µL of solution containing 20% sodium dodecyl sulfate and 50% dimethylformamide was added, followed by incubation at 37°C for 1 h and shaking on a shaker for 30 min. Absorbance at 580 nm was measured using a Synergy H1 plate reader. The three‐parameter nonlinear regression fitting in GraphPad Prism version 10 was used to calculate EC_50_ values.

For cytotoxicity assays without PBMCs, PCa cells were seeded at a density of 10^5^ cells per well in 96‐well plates and incubated with 5 nM of anti‐CD3‐DUPA ARC‐BsC for 48 h. The medium was replaced with 90 µL of fresh medium and 10 µL MTT (12 mM). The plates were incubated for 3 h at 37°C, followed by additions of 100 µL of 20% SDS 50% DMF solution for solubilization. Absorbance at 580 nm was measured using a Synergy H1 plate reader. PBS‐ and paclitaxel (5 µM)‐treated wells were included as 100% and 0% viability, respectively.

### 5.15 In Vitro T‐Cell Activation Assays

Prostate cancer cells (7 × 10^4^ cells per well) were mixed with human PBMCs (7 × 10^5^ cells per well) in 24‐well plates with a total volume of 0.5 mL. Wells with only human PBMCs were included as controls. Wells were treated in duplicates with 150 pM of anti‐CD3‐DUPA ARC BsC, a mixture of anti‐CD3 Fab HC‐CD38 and 2′ Cl‐araNAD^+^‐DUPA (molar ratio 1:1), or PBS for 48 h. Then, media and PBMCs were collected for analyzing levels of secreted interferon‐gamma (IFN‐γ) and granzyme B, and expressed T‐cell activation markers.

IFN‐γ and granzyme B secreted into media were measured using corresponding DuoSet ELISA kits by following manufacturer's protocols. At the last step, 100 µL of QuantaBlu fluorogenic substrates were added, followed by measuring fluorescence intensity (excitation wavelength: 325 nm; emission wavelength: 420 nm) using a Synergy H1 plate reader.

Collected PBMCs were resuspended in 100 µL of 2% FBS in PBS and placed in round bottom 96‐well plates. Each well was stained with FITC anti‐human CD3, PE anti‐human CD25, and APC anti‐human CD69 antibodies for 30 min at 4°C. Cells were then washed three times with 250 µL of 2% FBS in PBS and analyzed by a BD Fortessa X20 flow cytometer. Data were processed with FlowJo version 10 software. T cells were gated out using anti‐CD3 signals, and CD25‐ or CD69‐positive T cell percentages out of all T cells were calculated according to anti‐CD25 or anti‐CD69 signals.

### 5.16 In Vivo Efficacy and Toxicity

NGS mice (5 weeks, male, n = 5) were subcutaneously implanted with C4‐2 cells (5 × 10^6^ cells per mouse) in 50% Matrigel in right flanks. Freshly thawed PBMCs in RPMI 1640 medium were added into 6‐well cell culture plates pre‐coated for 3 h at 37°C with 1 mL of anti‐CD3 antibody (5 µg mL^−1^) at a density of 2 × 10^6^ cells mL^−1^, followed by additions of 10% FBS, anti‐CD28 antibody (2 µg mL^−1^), and recombinant human interleukin‐2 (IL‐2) (100 IU mL^−1^). After 48‐h activation at 37°C with 5% CO_2_, cells were transferred to antibody‐free flasks with IL‐2 supplements (100 IU mL^−1^) for in vitro expansion. On day 22 when tumors reached ∼100 mm^3^, human PBMCs were resuspended in PBS and injected into mice (2 × 10^7^ cells per mouse) via intraperitoneal injection. On day 23–31, mice received daily intravenous injections of anti‐CD3‐DUPA ARC‐BsC (1 mg kg^−1^) or PBS. Tumor size and weight were measured every 2–3 day. Tumor volume was calculated by the following equation using the length and width measured by caliper, volume = length × width ^2^/2.

At the end of the study, mice were euthanized, and blood, tumor, and major organs were harvested for each treatment group. Tumor fragments were embedded in O.C.T. formulation and frozen on dry ice for cryosectioning. To prepare single‐cell suspension, tumor and spleen samples were treated with DNase I and collagenase for 30 min at 37°C, cut into small pieces, and ran through 40 µm cell strainers. Cells were resuspended in 2% FBS in PBS and stained with PerCP/Cyanine5.5 anti‐human CD45 and FITC anti‐human CD3 antibodies for 30 min on ice. Samples were analyzed using a BD Fortessa X20 flow cytometer. Data were processed with FlowJo version 10 software. Cells were gated for CD45‐positive cells. Percentages of CD3‐positive cells were then calculated.

### 5.17 Immunofluorescence Imaging

Seven‐µm cryosections of tumors on slides were dried in a 37°C incubator for 20 min and submerged in PBS for 10 min at room temperature to dissolve O.C.T. Sections were then fixed with 4% PFA for 15 min, washed with PBST, and blocked in 3% BSA for 30 min. Slides were wiped carefully and stained with mouse anti‐human CD3 (0.5 µg mL^−1^) antibody overnight at 4°C. The next day, slides were washed three times with PBST and stained with Alexa Fluor 488 goat anti‐mouse IgG (10 µg mL^−1^) and Hoechst 33342 (10 µg mL^−1^) for 1 h at room temperature in the dark. Slides were then washed three times with PBST. Images were acquired by a Leica SP8 confocal laser scanning microscope (Leica Microsystems, IL)

### 5.18 Creatinine Colorimetric Assays

Collected plasma (20 µL) was mixed with 20 µL of 1.2 m trichloroacetic acid (TCA) solution, followed by centrifugation at 16000×g for 5 min. The supernatant (30 µL) was collected and mixed with 60 µL of working solution that was prepared by mixing 38 mM picritic acid and 1.2 m NaOH at a volume ratio 1:1. Standard solutions (30 µL) (creatinine at 10 different concentrations through threefold serial dilutions beginning at 1.2 mg mL^−1^) were also mixed with 60 µL of the working solution. Resulting solutions were incubated at room temperature for 20 min in the dark. Absorbance at 500 nm was measured using a Synergy H1 plate reader. Concentrations of creatinine in plasma samples were calculated using the standard curve.

### 5.19 ALT Activity Assays

Collected plasma (5 µL) and standard solutions (5 µL) (sodium pyruvate at 8 different concentrations through twofold serial dilutions beginning at 1 mM) were added to clear 96‐well plates. After additions of substrate solution (25 µL) (0.2 mM alanine, 2 mm 2‐oxoglutarate, pH 7.4) and subsequent incubation at 37°C for 20 min, 1 mm 2, 4‐dinitrophenylhydrazine in 1 m HCl (25 µL) was added into wells. Plates were incubated for 20 min in the dark and then added with 0.5 M NaOH (250 µL). Absorbance at 510 nm was measured using a Synergy H1 plate reader. Amounts of pyruvate generated were calculated from the standard curve. ALT activity was reported in U L^−1^, where 1 mU ALT generates 1 nmol of pyruvate per minute.

### 5.20 Statistical Analysis

In vivo tumor growth between two groups over time was statistically analyzed using a mixed‐effects model with repeated measures in GraphPad Prism version 10. All other statistical analyses were performed using unpaired t‐test in GraphPad Prism version 10. Data are shown as mean ± SD.

## Author Contributions

Y.Z. designed research. S.H.K., A.J.A., L.Z., G.K., T.O.H., Z.Z., and B.B.K. performed research. S.H.K., A.J.A., and Y.Z. analyzed data. S.H.K., A.J.A., and Y.Z. wrote the manuscript.

## Funding

NIH grants R35GM137901 (to Y. Z.), R01EB031830 (to Y. Z.), and R01CA276240 (to Y. Z.).

## Conflicts of Interest

The authors declare no conflicts of interest.

## Supporting information




**Supporting File**: advs73399‐sup‐0001‐SuppMat.docx.

## Data Availability

The data that support the findings of this study are available from the corresponding author upon reasonable request.
