# Supplementary material for: Synthesis of Bispecific Conjugates by ADP‐Ribosyl Cyclases
Source: Adv Sci (Weinh). 2025 Dec 21;13(13):e18329. doi: 10.1002/advs.202518329 (PMC12955874; doi:10.1002/advs.202518329)
Supplement: Supplementary file 1 — Supporting File: advs73399‐sup‐0001‐SuppMat.docx. [file ADVS-13-e18329-s001.docx]

Supporting Information

Synthesis of Bispecific Conjugates by ADP-Ribosyl Cyclases

Sunny H. Kim^1#^, Arshad J. Ansari^1#^, Lei Zhang^1^, Guoyun Kao^1^, Thuc Oanh Hoang^1^, Zeyu Zhang^1^, Srinivasarao Singireddi^1^, Benjamin B. Katz^2^, and Yong Zhang^1,3,4,5*^

^1^Department of Pharmacology and Pharmaceutical Sciences, Alfred E. Mann School of Pharmacy and Pharmaceutical Sciences, University of Southern California, Los Angeles, CA 90089, USA

^2^Department of Chemistry, University of California, Irvine, Irvine, CA 92697, USA

^3^Department of Chemistry, Dornsife College of Letters, Arts and Sciences, University of Southern California, Los Angeles, CA 90089, USA

^4^Norris Comprehensive Cancer Center, University of Southern California, Los Angeles, CA 90089, USA

^5^Research Center for Liver Diseases, University of Southern California, Los Angeles, CA 90089, USA

^#^ These authors contributed equally to this work

^*^Email: [yongz@usc.edu](mailto:yongz@usc.edu)

**
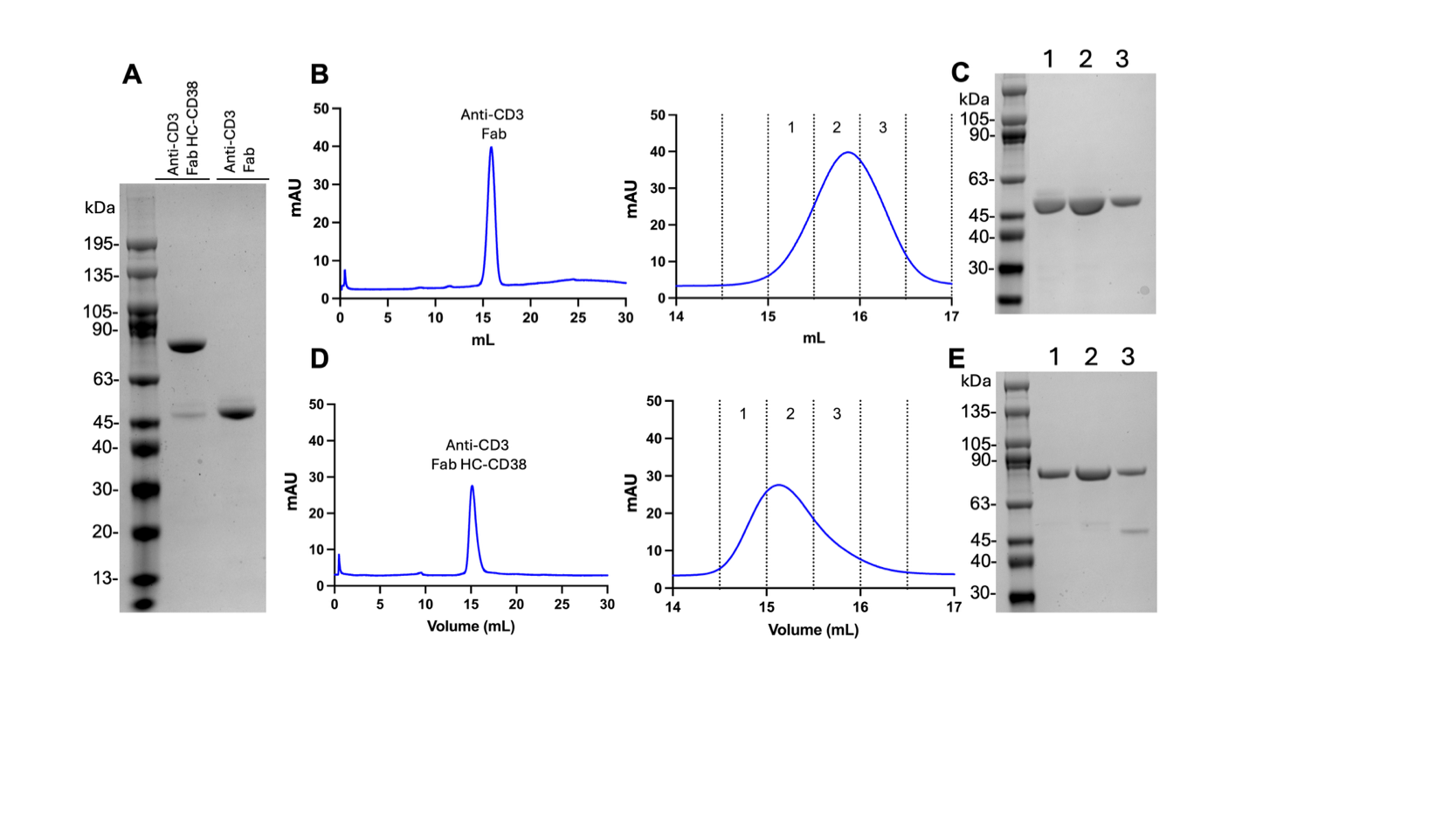
**

Figure S1. Gel filtration chromatography of purified antibodies. (A) Coomassie-stained SDS-PAGE gel of non-reduced antibodies prior to gel filtration chromatographic analysis. (B)-(D) Gel filtration chromatograms and Coomassie-stained SDS-PAGE gels of anti-CD3 Fab (B) and (C) and anti-CD3 Fab HC-CD38 (D) and (E). Fractions 1-3 containing non-reduced antibodies were analyzed by SDS-PAGE gels.

**
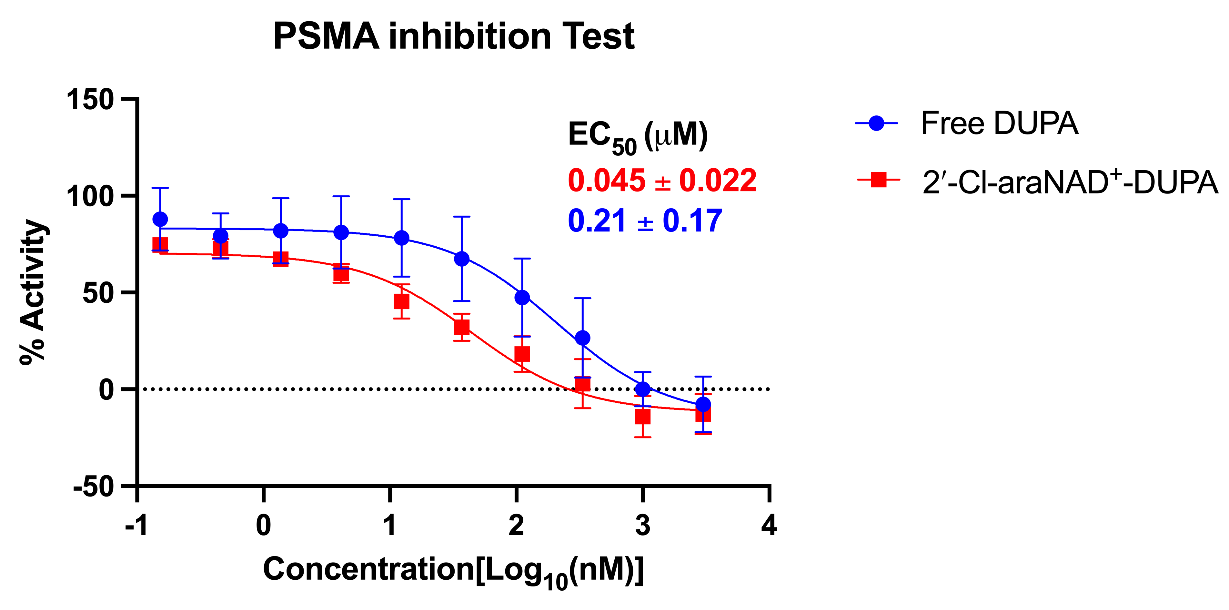
**

Figure S2. Inhibition activity of 2ʹ-Cl-araNAD^+^-DUPA. Recombinant PSMA was incubated with its substrate NAAG in the presence of free DUPA or 2ʹ-Cl-araNAD^+^-DUPA at varied concentrations. The formation of glutamic acid product was measured with a fluorescent derivation reagent, OPA. The activity was normalized based on PSMA activity in the absence of inhibitor (100%) or substrate (0%).


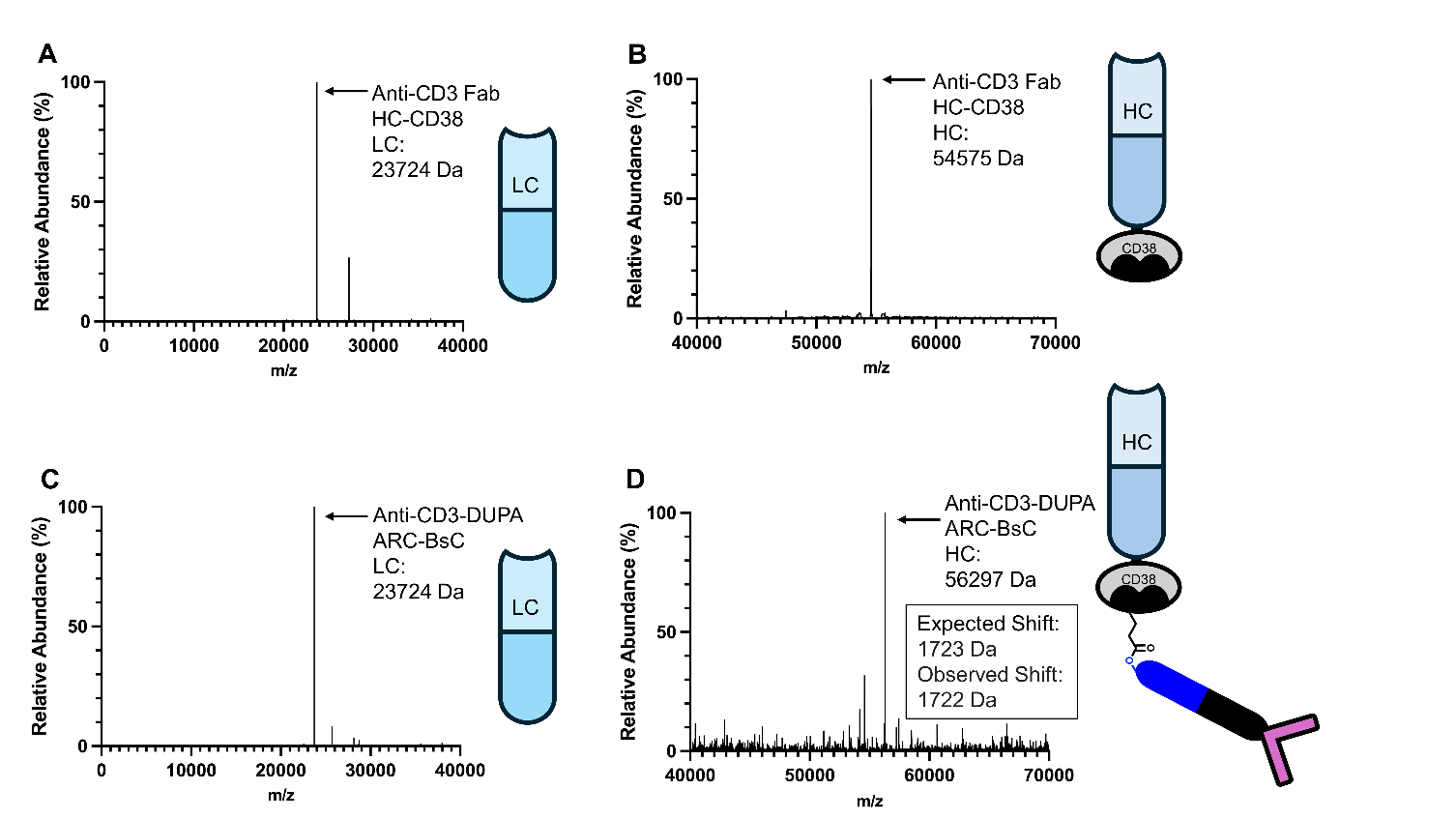


Figure S3. Mass spectra of unconjugated anti-CD3 Fab HC-CD38 and anti-CD3-DUPA ARC-BsC. (A) and (B) Light chain (LC) and heavy chain (HC) of anti-CD3 Fab HC-CD38. (C) and (D) LC and HC of anti-CD3-DUPA ARC-BsC. Samples were reduced by DTT prior to the analysis.


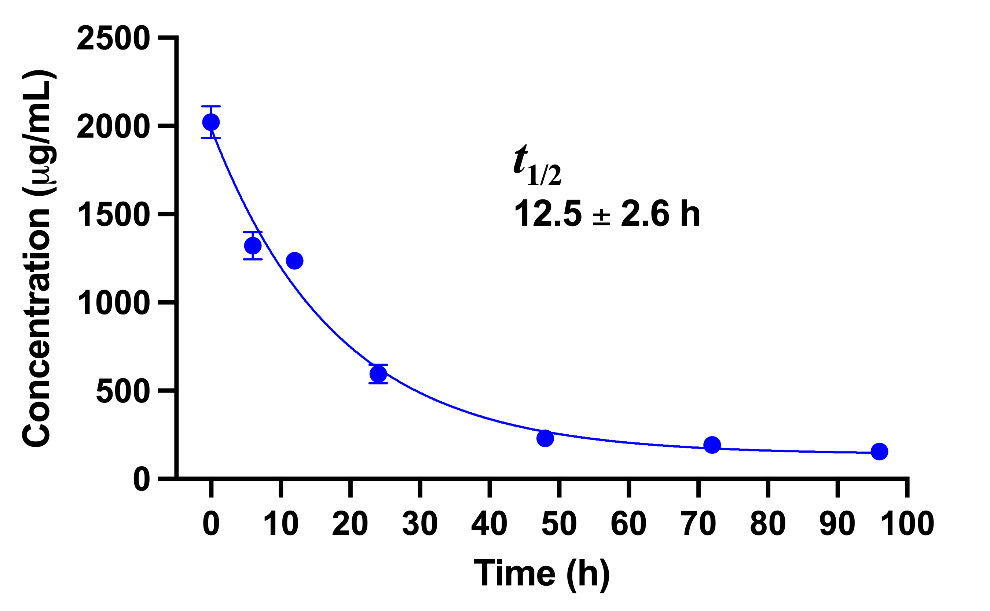


Figure S4. Serum stability of DUPA conjugation. Anti-CD3-DUPA ARC-BsC was incubated at 37°C in 100% FBS for up to 96 h. Intact anti-CD3-DUPA ARC-BsC at 0, 6, 12, 24, 48, 72, 96 h were quantified based on the binding to recombinant PSMA via ELISA assays.


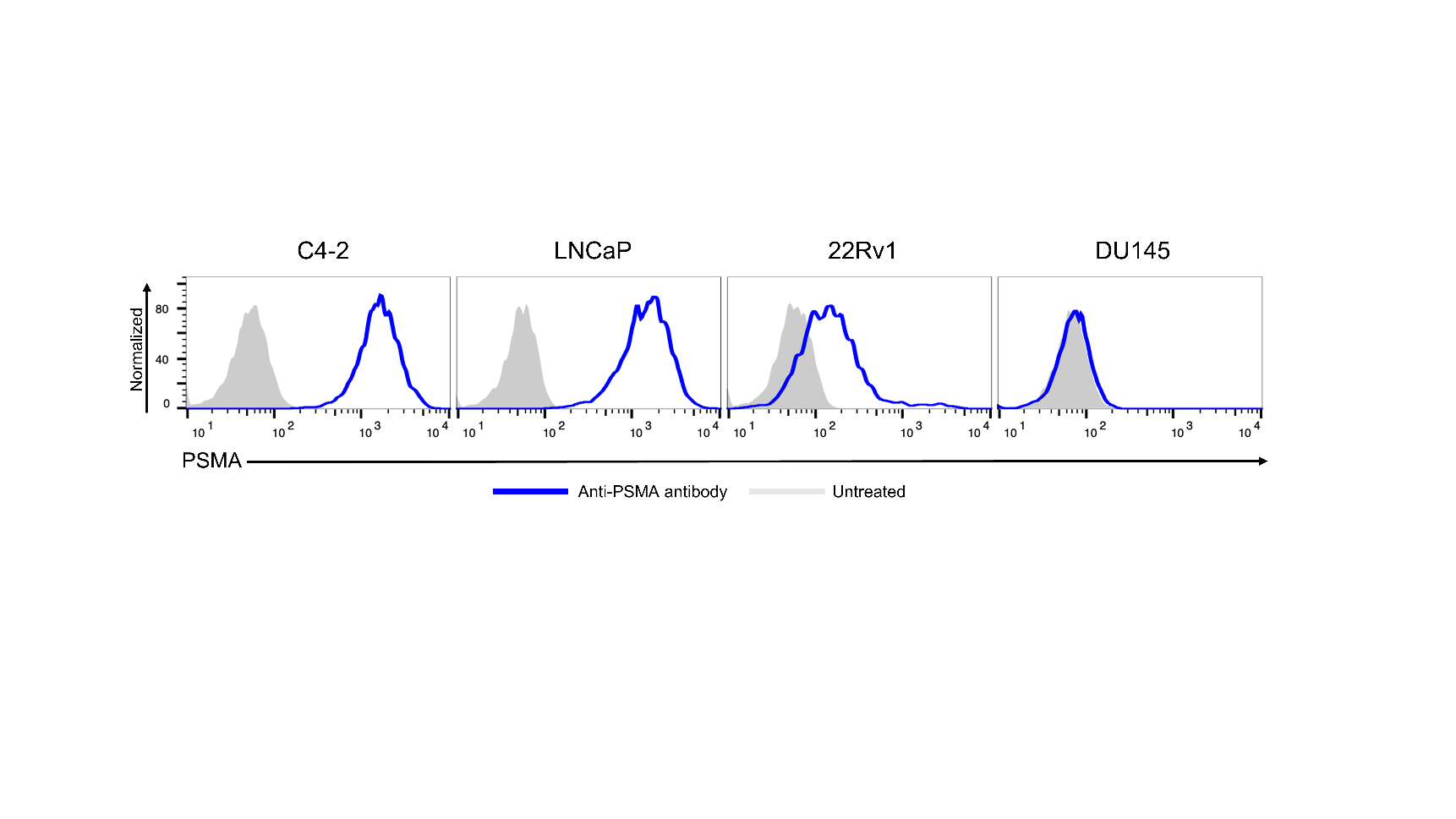


Figure S5. Flow cytometric analysis of expression levels of PSMA on PCa cell lines.


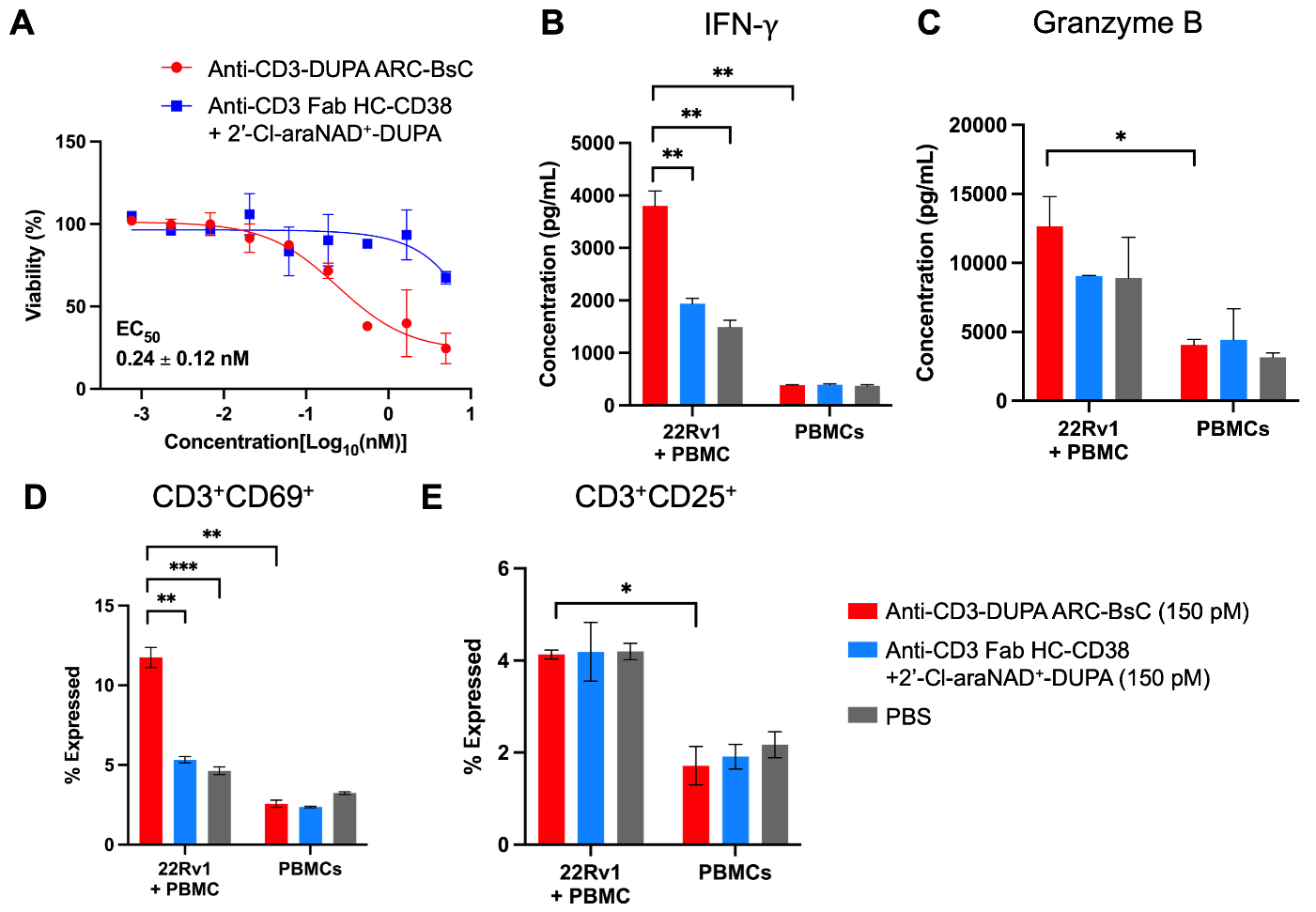


Figure S6. *In vitro* cytotoxicity of anti-CD3-DUPA ARC-BsC for 22Rv1 cells in the presence of human PBMCs. 22Rv1 cells were mixed with human PBMCs at a ratio of 1:10 and incubated for 48 h with anti-CD3-DUPA ARC-BsC at varied concentrations. After removing PBMCs, the viability of PCa cells were measured. A mixture of anti-CD3 Fab HC-CD38 and 2ʹ-Cl-araNAD^+^-DUPA conjugate (molar ratio 1:1) was included as controls.


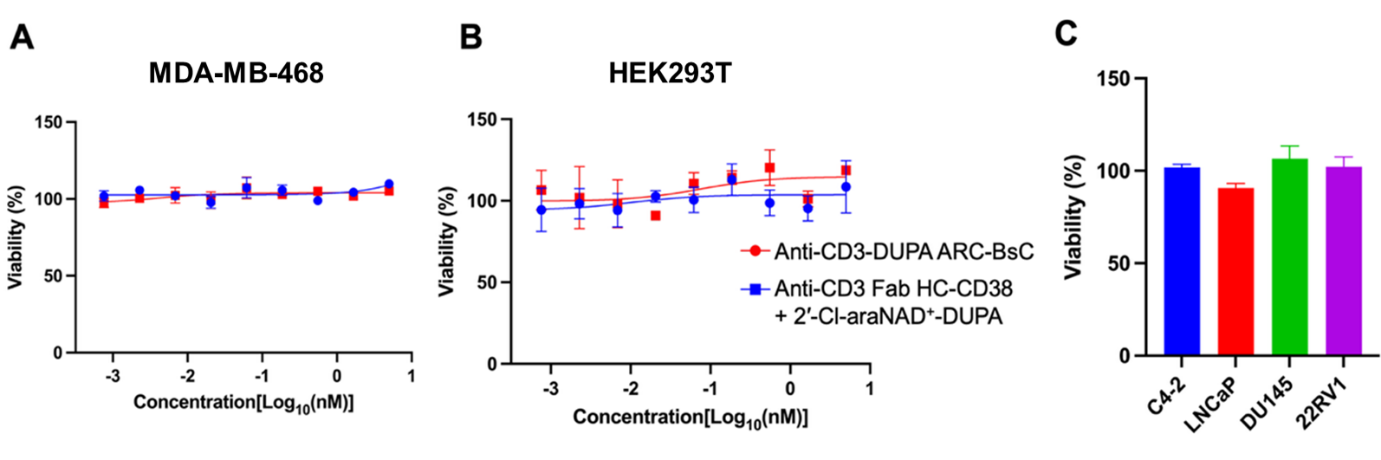


Figure S7. *In vitro* cytotoxicity of anti-CD3-DUPA ARC-BsC. (A) and (B) *In vitro* cytotoxicity of anti-CD3-DUPA ARC-BsC for MDA-MB-468 (A) and HEK293T (B) cells in the presence of human PBMCs. MDA-MB-468 or HEK293T cells were mixed with human PBMCs at a ratio of 1:10 and incubated for 48 h with anti-CD3-DUPA ARC-BsC at various concentrations. After removal of PBMCs, the viability of MDA-MB-468 or HEK293T cells were measured. A mixture of anti-CD3 Fab HC-CD38 and 2ʹ-Cl-araNAD^+^-DUPA conjugate (molar ratio 1:1) was included as controls. (C) *In vitro* cytotoxicity of anti-CD3-DUPA ARC-BsC for PCa cells with varied levels of PSMA expression in the absence of PBMCs. PCa cells were incubated with 5 nM of anti-CD3-DUPA ARC-BsC for 48 h at 37°C with 5% CO_2_. The cell viability was then measured via MTT assays and normalized with PBS-treated cells (100% viability) and paclitaxel (5 μM)-treated cells (0% viability).


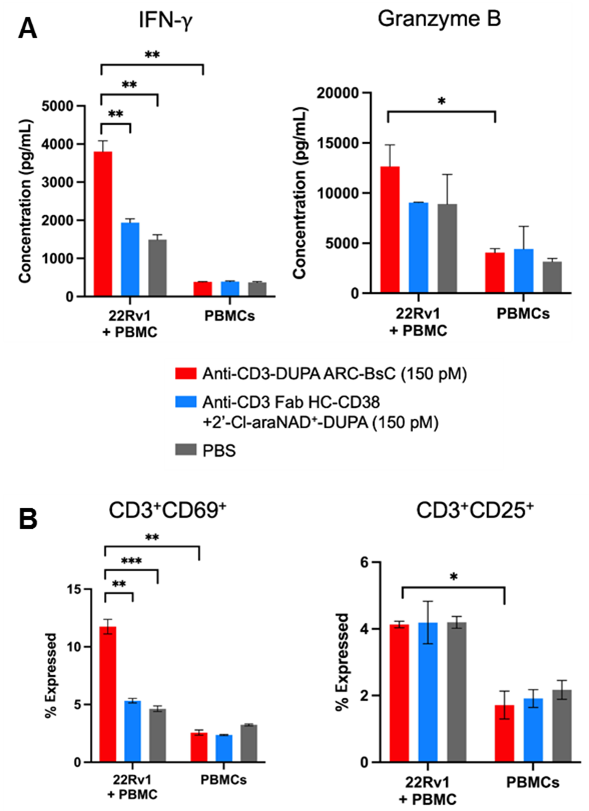


Figure S8. *In vitro* T-cell activation by anti-CD3-DUPA ARC-BsC for 22Rv1 cells. (A) Secretion levels of IFN-γ and granzyme B. (B) Expression levels of CD69 and CD25. Human PBMCs without and with 22Rv1 cells (ratio 10:1) were incubated for 48 h in the presence of anti-CD3-DUPA ARC-BsC (150 pM), a mixture of anti-CD3 Fab HC-CD38 and 2ʹ-Cl-araNAD^+^-DUPA conjugate (150 pM, molar ratio 1:1), or PBS. Secreted IFN-γ and granzyme B were measured by ELISA. Expression of CD69 and CD25 on T cells were analyzed by flow cytometry. * p < 0.1; ** p < 0.01; *** p < 0.001.

**Chemical synthesis of 2′-Cl-araNAD^+^-DUPA and compound characterization**

Commercial reagents were used as received unless otherwise indicated. Solvents were used as received from commercial suppliers. The reactions were conducted under anhydrous conditions in flasks that were oven-dried for 30 min under a dry nitrogen atmosphere. Flash column chromatography was performed using 230-400 mesh silica gel (Sigma-Aldrich, St. Louis, MO). Thin-layer chromatography (TLC) was performed using silica gel plates (Sigma-Aldrich, GF254) and analyzed under 254 nm UV light using the diluted samples. High-resolution mass spectra (HRMS) were obtained using Thermo Fisher Q-Exactive Orbitrap (LC/MS/MS) at the Multi-Omics Mass Spectrometry Core of the University of Southern California. ^1^H NMR spectra were recorded using an Oxford AM-400 spectrometer in CDCl_3_, CD_3_OD, or D_2_O. The coupling constants, J, are shown in hertz. ^13^C NMR spectra were recorded on an Oxford AM-400 spectrophotometer (100 MHz) with a complete proton-decoupling spectrophotometer (CD_3_OD: 49.0 ppm).

1. **Synthesis of 2-[3-dicarboxy propyl]-ureido] pentanedioic acid (DUPA).**

**Synthesis of Int-1 (Step 1)**. In a solution of di-tert-butyl *L*-glutamate hydrochloride (1 eq.) and 4-dimethylaminopyridine (DMAP, 16.5 mg, 0.135 mmol, 0.04 eq.) in anhydrous dichloromethane (DCM) (25 mL) under inert atmosphere, Et_3_N (1.18 mL, 8.5 mmol, 2.5 eq.) was added dropwise at room temperature (rt), forming a white precipitate. The suspension was cooled to 0^o^C, and 1,1'-carbonyldiimidazole (CDI, 604 mg, 3.7 mmol, 1.1 eq.) in anhydrous DCM (25 mL) was added dropwise. The resulting mixture was then stirred overnight at rt. DCM (25 mL) was added, and the organic phase was extracted with saturated NaHCO_3_, followed by two washes with water and saturated NaCl. The organic fraction was concentrated in vacuo to afford **Int-1** (1.1 g) in the form of a yellowish gel. The product was used in the next reaction step without further purification.

**Synthesis of 1A (Step 2)**. A solution of di-tert-butyl (1*H*-imidazole-1-carbonyl)-*l-*glutamate (**Int-1**) (1.1 g, 3.11 mmol, 1.0 eq.) and H-*L*-Glu(OBn)-O*^t^*Bu.HCl (0.91 g, 3.11 mmol, 1.0 eq.) in DCM (15 mL) was cooled to 0°C and afterwards treated dropwise with Et_3_N (0.90 mL, 6.22 mmol, 2.0 eq.). The reaction mixture was initially stirred at 0°C to reach rt and then stirred overnight. After concentrating the mixture in vacuo, the residue was re-dissolved in MeOH (5 mL) and purified by flash column chromatography (silica, 40-50% EtOAc in petroleum (pet.) ether), yielding **1A** (1.85 g, 3.19 mmol, 85%) as a colorless oil. **^1^H NMR (400 MHz, CDCl_3_) δ** 7.36-7.27 (m, 5H), 5.09 (d, *J* = 1.9 Hz, 2H), 4.33 (ddd, *J* = 20.4, 8.0, 5.0 Hz, 2H), 2.43 (ddd, *J* = 13.1, 9.2, 6.3 Hz, 1H), 2.29 (ddd, *J* = 10.7, 9.2, 6.5 Hz, 2H), 2.14 (dddd, *J* = 14.2, 9.3, 6.5, 5.0 Hz, 1H), 2.09-1.98 (m, 2H), 1.96-1.77 (m, 2H), 1.43 (d, *J* = 2.9 Hz, 18H), 1.41 (s, 9H). **^13^C NMR (101 MHz, CDCl_3_) δ** 172.94, 172.50, 171.83, 171.70, 156.90, 135.81, 128.52, 128.21, 128.19, 82.19, 82.11, 80.57, 66.40, 60.41, 53.03, 52.96, 31.50, 31.47, 30.32, 28.37, 28.30, 28.04, 27.96, 21.05, 14.18. **HRMS (ESI) for C_30_H_46_N_2_O_9_ (M+K)^+^**: Calcd.: 617.2840; Obs: 617.2839.

**Synthesis of 1B (Step 3).** For benzyl (Bn) deprotection, 5-benzyl 1-(tert-butyl) (((S)-1,5-di-tert-butoxy-1,5-dioxopentan-2-yl)carbamoyl)-L-glutamate (**1A**) (1.85 g, 3.19 mmol, 1.0 eq.) and 10% palladium on carbon (340 mg, 0.32 mmol, 0.1 eq., w/w) were dissolved in MeOH/triethylsilane (1:1, 20 mL) in a sealed vial. The reaction mixture was stirred overnight at rt, filtered through Celite, and concentrated in vacuo. The resulting residue was purified by flash column chromatography (silica, 50-60% EtOAc in pet. ether) to afford **1B** (1.40 g, 2.87 mmol, 90%) as a colorless, hygroscopic solid. **^1^H NMR (400 MHz, CDCl_3_) δ** 4.46 (dd, *J* = 8.6, 4.8 Hz, 1H), 4.30 (dd, *J* = 9.8, 4.2 Hz, 1H), 2.46-2.36 (m, 2H), 2.35-2.24 (m, 2H), 2.18-2.02 (m, 2H), 1.95-1.79 (m, 2H), 1.47 (s, 9H), 1.44 (s, 9H), 1.42 (s, 9H). **^13^C NMR (101 MHz, CDCl_3_) δ** 175.87, 172.49, 171.69, 158.06, 82.75, 82.15, 80.69, 53.49, 52.99, 31.49, 30.46, 28.44, 28.05, 28.00, 27.96, 27.73. **HRMS (ESI) for C_23_H_40_N_2_O_9_ (M+Na)^+^:** 511.2632; Obs: 511.2638.

1. **Synthesis of linker.**

**Synthesis of 2A (Step 1**). To a solution of 1,5-dichloro-2,4-dinitrobenzene (1.0 g, 4.23 mmol, 1.0 eq.) in ethanol (20.0 mL) were added *N*-Boc-1,6-hexanediamine (1.0 g, 4.66 mmol, 1.1 eq.), and stirred for 12 h at rt as monitored by TLC. The solvent was then removed under reduced pressure and the residue was purified by flash column chromatography using a 4:1 pet. ether in EtOAc as the eluent to afford **2A** (1.32 g, 3.1 mmol, 75%) as a yellow oil. **^1^H NMR (400 MHz, CDCl_3_) δ** 9.05 (s, 1H), 8.38 (t, *J* = 5.2 Hz, 1H), 6.93 (s, 1H), 3.36 (td, *J* = 7.1, 5.2 Hz, 2H), 3.12 (t, *J* = 6.9 Hz, 2H), 1.82-1.73 (m, 2H), 1.55-1.45 (m, 5H), 1.44 (d, *J* = 1.4 Hz, 9H), 1.41 (d, *J* = 6.9 Hz, 1H). **^13^C NMR (101 MHz, CDCl_3_) δ** 156.04, 146.75, 135.94, 134.47, 128.91, 127.00, 116.05, 43.55, 30.00, 28.51, 28.40, 26.53, 26.26. **HRMS (ESI) for C_17_H_25_ClN_4_O_6_ (M+Na)^+^:** Calcd.: 439.1360; Obs: 439.1349.

**Synthesis of 2B (Step 2)**. To a solution of tert-butyl (6-((5-chloro-2,4-dinitrophenyl)amino)hexyl)carbamate (1.32 g, 3.1 mmol, 1.0 eq.) in DCM (20.0 mL) under inert atmosphere were cooled to 0^o^C and added 4 N HCl in dioxane (8 mL) and reaction mixture was stirred at 0^o^C for 24 h. The reaction was monitored by TLC. The solvent was then removed under reduced pressure, and the residue was washed with ether to afford **2B** (0.90 g, 90 %) as a yellow solid. **^1^H NMR (400 MHz, CD_3_OD) δ** 8.96 (s, 1H), 7.20 (s, 1H), 3.46 (dt, *J* = 10.1, 7.1 Hz, 2H), 3.07-2.90 (m, 2H), 1.73 (dq, *J* = 23.0, 7.2 Hz, 4H), 1.50 (dq, *J* = 8.1, 4.0 Hz, 5H), 1.42 (s, 2H). **^13^C NMR (101 MHz, CD_3_OD) δ** 146.77, 134.73, 128.87, 126.27, 116.14, 116.12, 42.73, 39.21, 27.98, 27.36, 27.07, 26.01, 25.72. **HRMS (ESI) for C_12_H_18_ClN_4_O_4_ (M+H)^+^:** Calcd.: 317.1017; Obs: 317.1030.

**Synthesis of 3A** (**Step 1**). To a solution of 2-butyne-1,4-diol (5 g, 58.3 mmol, 1.0 eq.) in DMSO:H_2_0 (4:1) at 0^o^C were add KOH pellets (12.8 g, 233 mmol g, 4.0 eq.). The reaction mixture was stirred for 30 min. 1,5-dibromopentane (31.4 ml, 233.2 mmol, 4.0 eq.) was added via a syringe to the reaction mixture. The reaction mixture was stirred overnight. The reaction mixture was then poured into ice/water. Extract the product 3 times with ether and combined organic layer washed with water and brine. The combined organic layers were then dried over Na_2_SO_4_. The organic solution was concentrated. The solution was purified using flash chromatography on silica gel (10% EtOAc in pet. ether) to afford **3A** (13.3 mg, 60 %) as a colorless oil. **^1^H NMR (400 MHz, CDCl_3_) δ** 4.14 (s, 4H), 3.48 (t, *J* = 6.3 Hz, 4H), 3.38 (t, *J* = 6.8 Hz, 4H), 1.90-1.81 (m, 4H), 1.61-1.55 (m, 4H), 1.49 (tdd, *J* = 9.0, 4.8, 1.7 Hz, 4H). **^13^C NMR (101 MHz, CDCl_3_) δ** 82.28, 77.43, 77.11, 76.79, 69.74, 58.33, 33.73, 32.52, 28.66, 24.84. **HRMS (ESI) for C_14_H_25_Br_2_O_2_ (M+H)^+^:** Calcd.:383.0221; Obs: 383.0212.

**Synthesis of 3B** (**Step 2**). To a solution of 1,4-bis((5-bromopentyl)oxy)but-2-yne **3A** (3 g, 13.0 mmol, 1.0 eq.) in DMF, was added tetrabutylammonium bromide-TBAB (16.87 g, 52.0 mmol, 4.0 eq.), followed by potassium carbonate (7.2 g, 52.0 mmol, 4.0 eq.) and phthalimide (5.77 g, 39.2 mmol, 3.0 eq.). The reaction mixture was stirred for 12 h at 80°C. Reaction was monitored using TLC and mass spectrometry. After completion of the reaction, reaction mixture was cooled and extracted with methylene chloride (2 × 25 mL). The organic layer was dried over Na_2_SO_4_, filtered, and evaporated to dryness. The residue was purified by flash chromatography using 20% EtOAc in pet. ether as an eluent to afford **3B** (2.83 g, 70 %) as a colorless solid. **^1^H NMR (400 MHz, CDCl_3_) δ** 7.82 (dd, *J* = 5.4, 3.1 Hz, 4H), 7.70 (dd, *J* = 5.4, 3.0 Hz, 4H), 4.14 (s, 4H), 3.67 (t, *J* = 7.3 Hz, 4H), 3.47 (t, *J* = 6.5 Hz, 4H), 1.73-1.66 (m, 4H), 1.64-1.58 (m, 5H), 1.46-1.36 (m, 4H). **^13^C NMR (101 MHz, CDCl_3_) δ** 168.43, 133.86, 132.13, 123.16, 82.26, 69.80, 58.30, 37.89, 29.06, 28.39, 23.46. **HRMS (ESI) for C_30_H_32_N_2_O_6_ (M+K)^+^: Calcd.:** 555.1897; Obs: 555.1897.

**Synthesis of 3C** (**Step 3**). To a solution of 2,2'-((but-2-yne-1,4-diylbis(oxy))bis(pentane-5,1-diyl))bis(isoindoline-1,3-dione) **3B** (6.5 g, 1.0 eq.) in ethanol (200 mL) was added NH_2_NH_2_.H_2_O (4 mL) at rt. The resulting mixture was stirred at 80°C for 24 h, and the reaction was monitored using mass spectrometry and TLC. After the completion of the reaction, a white precipitate was formed and filtered using celite, and the filtrate was concentrated and purified by flash column chromatography using 5-10% methanol in DCM as the eluent to afford **3C** (2.4 g, 75%) as a colorless oil. **^1^H NMR (400 MHz, CD_3_OD) δ** 4.14 (s, 4H), 3.49 (t, *J* = 6.4 Hz, 4H), 2.64 (s, 4H), 1.60-1.54 (m, 6H), 1.49 (tt, *J* = 7.1, 1.5 Hz, 5H), 1.37 (td, *J* = 5.6, 2.6 Hz, 4H). **^13^C NMR (101 MHz, CD_3_OD) δ** 128.97, 81.92, 70.30, 70.28, 69.90, 69.41, 66.03, 57.51, 40.77, 31.39, 31.36, 29.13, 28.94, 26.12, 23.17, 23.15, 23.11. **HRMS (ESI) for C_14_H_29_N_2_O_2_ (M+H)^+^:** Calcd.: 257.2229; Obs: 257.2233.

**Synthesis of 3D** (**Step 4**). 5,5'-(but-2-yne-1,4-diylbis(oxy))bis(pentan-1-amine) **3C** (2.0 g, 0.0078 mmol, 1.0 eq.) was dissolved in THF with water added if needed and cooled to 0°C. Boc-ON (1.921 g, 0.0078 mmol, 1.0 eq.) was then added, followed by Et_3_N (1.75 mL, 0.0117 mmol, 1.5 eq.). The reaction mixture was stirred overnight and monitored by mass spectrometry. After completion of the reaction, the solvent was evaporated under reduced pressure and the residue was purified by flash column chromatography using 5-10% methanol in DCM as the eluent to afford **3D** (2.2 g, 80%) as a colorless oil. **^1^H NMR (400 MHz, CDCl_3_) δ** 4.62 (s, 1H), 4.14 (s, 4H), 3.47 (td, *J* = 6.5, 2.9 Hz, 4H), 3.39 (td, *J* = 6.6, 2.9 Hz, 2H), 3.07 (t, *J* = 6.7 Hz, 2H), 1.60-1.54 (m, 6H), 1.47 (t, *J* = 7.2 Hz, 4H), 1.41 (s, 9H), 1.39 -1.34 (m, 4H). **^13^C NMR (101 MHz, CDCl _3_) δ** 155.99, 129.38, 82.27, 82.25, 69.99, 69.93, 66.44, 58.30, 30.28, 29.81, 29.47, 29.36, 29.25, 29.14, 28.40, 23.46, 23.39. **HRMS (ESI) for C_19_H_37_N_2_O_4_ (M+H)^+^:** Calcd.: 357.2753; Obs: 357.2769.

**Synthesis of 4A (Step 5).** N1-(5-chloro-2,4-dinitrophenyl)hexane-1,6-diamine **2B** (800 mg, 2.53 mmol, 1.0 eq.) was dissolved in 20 mL EtOH and tert-butyl (5-((4-((5-aminopentyl)oxy)but-2-yn-1-yl)oxy)pentyl)carbamate **3D** (1.35 g, 3.79 mmol, 1.5 eq.) was added, followed by K_2_CO_3_ (500 g, 3.03 mmol, 1.2 eq.). The resulting mixture was stirred at 60°C for 24-36 h and the reaction was monitored by TLC and mass spectrometry. The solvent was then removed under reduced pressure, and the residue was purified by flash column chromatography using 5-10% methanol in DCM as the eluent to afford **4A** (0.88 g, 55%) as a yellow solid. **^1^H NMR (400 MHz, CDCl_3_) δ** 9.10 (s, 1H), 8.25 (t, *J* = 4.8 Hz, 2H), 4.17 (d, *J* = 9.1 Hz, 4H), 4.03 (dd, *J* = 8.5, 4.2 Hz, 1H), 3.53 (t, *J* = 6.3 Hz, 2H), 3.48 (t, *J* = 6.4 Hz, 2H), 3.40 (t, *J* = 6.6 Hz, 1H), 3.25 (t, *J* = 6.0 Hz, 4H), 3.10 (d, *J* = 6.8 Hz, 5H), 1.84-1.76 (m, 6H), 1.67 (q, *J* = 6.9 Hz, 3H), 1.58 (d, *J* = 7.1 Hz, 4H), 1.53-1.48 (m, 6H), 1.42 (s, 9H), 1.37 (d, *J* = 7.1 Hz, 3H). **^13^C NMR (101 MHz, CDCl_3_) δ** 156.00, 148.48, 148.42, 129.48, 129.34, 129.27, 123.95, 123.73, 90.02, 82.42, 82.15, 79.05, 70.01, 69.72, 66.57, 66.46, 58.41, 58.30, 53.46, 43.31, 43.06, 40.49, 29.83, 29.70, 29.38, 29.15, 28.41, 28.22, 27.98, 26.36, 23.80, 23.41. **HRMS (ESI) for C_31_H_53_N_6_O_8_ (M+H)^+^:** Calcd.: 637.3935; Obs: 637.3941.

**Synthesis of 4B** (**Step 6**). To a solution of tert-butyl (5-((4-((5-((5-((6-aminohexyl)amino)-2,4-dinitrophenyl)amino)pentyl)oxy)but-2-yn-1-yl)oxy)pentyl)carbamate **4A** (350 mg, 0.55 mmol, 1.0 eq.) in DMF, was added propargyl-PEG4-NHS ester (216 mg, 0.60 mmol, 1.1 eq.), followed by Et_3_N (120 µL, 0.82 mmol, 1.5 eq.). The resulting mixture was stirred at rt for 24 h and monitored by mass spectrometry and TLC. The solvent was then removed under reduced pressure, and the residue was purified by flash column chromatography using 5-10% methanol in DCM as the eluent to afford **4B** (372 mg, 77%) as a yellow semi-solid. **^1^H NMR (400 MHz, CDCl_3_) δ** 9.21 (d, *J* = 0.7 Hz, 1H), 8.30 (d, *J* = 5.8 Hz, 1H), 5.61 (s, 1H), 4.18 (dd, *J* = 2.4, 0.7 Hz, 2H), 4.16 (dd, *J* = 7.9, 1.7 Hz, 4H), 3.69-3.60 (m, 14H), 3.52 (t, *J* = 6.2 Hz, 2H), 3.46 (d, *J* = 0.7 Hz, 13H), 3.31-3.21 (m, 6H), 3.08 (t, *J* = 7.0 Hz, 2H), 3.00 (d, *J* = 0.7 Hz, 2H), 2.57 (t, *J* = 5.6 Hz, 2H), 1.82-1.72 (m, 4H), 1.71-1.63 (m, 2H), 1.56 (qd, *J* = 9.1, 4.3 Hz, 6H), 1.50-1.45 (m, 4H), 1.45-1.39 (m, 11H), 1.39-1.33 (m, 2H). **^13^C NMR (101 MHz, CDCl_3_) δ** 171.67, 148.59, 130.39, 129.64, 124.01, 90.06, 82.10, 77.20, 74.62, 74.61, 70.56, 70.37, 70.16, 70.00, 69.68, 69.06, 67.30, 58.39, 53.42, 43.24, 39.11, 36.96, 29.68, 29.52, 29.14, 28.40, 28.23, 26.75, 26.49, 23.81. **HRMS (ESI) for C_43_H_71_N_6_O_13_ (M+H)^+^:** Calcd.: 879.5079; Obs: 879.5077.

**Synthesis of 4C** (**Step 7**). To a solution of tert-butyl (5-((4-((5-((2,4-dinitro-5-((16-oxo-4,7,10,13-tetraoxa-17-azatricos-1-yn-23-yl)amino)phenyl)amino)pentyl)oxy)but-2-yn-1-yl)oxy)pentyl)carbamate **4B** (300 mg, 1.0 eq.) in 10 mL DCM at 0^o^C, was added 4 N HCl in 2 mL dioxane. The reaction mixture was stirred at 0-5^o^C for 12 h and monitored using mass spectrometry. After completion of the reaction, solvent was evaporated using reduced pressure, followed by washing with diethyl ether and drying to afford **4C** (255 mg, 95%) as a yellow solid, which was used for the next step without further purification. **HRMS (ESI) for C_38_H_63_N_6_O_11_ (M+H)^+^:** Calcd.: 779.4555; Obs:779.4540.

1. **Synthesis of DUPA-linker conjugate.**

**Synthesis of 5A**. To a stirred solution of DUPA **1B** (96 mg, 0.19 mmol, 1.0 eq.), HATU (101.0 mg, 0.25 mmol, 1.3 eq.) and DMAP (7.1 mg, 0.058 mmol, 0.3 eq.) in DMF (0.5 mL), was added a solution of N-(6-((5-((5-((4-((5-aminopentyl)oxy)but-2-yn-1-yl)oxy)pentyl)amino)-2,4-dinitrophenyl)amino)hexyl)-4,7,10,13-tetraoxahexadec-15-ynamide **4C** (170 mg, 0.22 mmol, 1.2 eq.) and DIPEA (102 µL, 0.59 mmol, 3 eq.) in DMF (0.5 mL) at 0^o^C. The reaction mixture was then allowed to warm to rt and stirred at the same temperature until the reaction was completed (monitored by mass spectrometry). The reaction mixture was concentrated in vacuo, and the residue was purified by flash column chromatography using 5-10% methanol in DCM as the eluent to afford **5A** (208 mg, 83%) as a yellow semi-solid**. HRMS (ESI) for C_61_H_101_N_8_O_19_ (M+H)^+^:** Calcd.: 1249.7183; Obs: 1249.7176.

**Synthesis of 5B.** To a stirred solution of tri-tert-butyl (3S,7S)-27-((2,4-dinitro-5-((16-oxo-4,7,10,13-tetraoxa-17-azatricos-1-yn-23-yl)amino)phenyl)amino)-5,10-dioxo-17,22-dioxa-4,6,11-triazaheptacos-19-yne-1,3,7-tricarboxylate **5A** (100 mg, 1 eq.) in MeOH (2 mL), was added 8 mL TFA:H_2_O (4:1) at 0^o^C. The reaction was allowed to proceed at 5^o^C for 10-12 h until the reaction was completed (monitored by mass spectrometry). The reaction mixture was concentrated in vacuo, and the residue was washed with diethyl ether and concentrated to afford **5B** (75.28 mg, 87%) as a yellow solid, which was used for the next step without further purification. **HRMS (ESI) for C_49_H_76_N_8_O_19_ (M+Na)^+^:** Calcd.: 1103.5124; Obs: 1103.5115.

1. **Synthesis of 2′-Cl-araNAD^+^-DUPA.**

**Synthesis of 2′-Cl-araNAD^+^-DUPA**. To a solution of compound **5B** (10.00 mg, 0.0092 mmol) and CuSO_4_.5H_2_O (9.23 mg, 0.037 mmol, 4 eq.) in H_2_O (500 μL), was added a solution of 6-azido-2′-Cl-araNAD^+^ (7.04 mg, 0.0092 mmol, 1.0 eq.) prepared as previously reported along with THPTA (32 mg, 0.074 mmol, 8 eq.) and sodium-L-ascorbate (13.0 mg, 0.074 mmol, 8 eq.) at rt.(1) The reaction mixture was stirred at the same temperature until the reaction was completed (monitored by mass spectrometry). The reaction product was purified via HPLC (C18-A column, 150×4.6 mm, 5 µm) (mobile phase A: 0.1% formic acid (aq.); mobile phase B: 0.1% formic acid (aq.) in acetonitrile; flow rate = 1.0 mL min^-1^; 0-2 min: 0-4% B, 2-4 min: 4-10% B, 4-6 min: 10-20% B, 6-12 min: 20-50% B, 12-17 min: 50-100% B, and 17-20 min: 100-0% B) with detection of UV absorbance at 260 nm. Fractions containing the desired product were concentrated and lyophilized to yield 2′-Cl-araNAD^+^-DUPA (7.6 mg, 48%) as a colorless solid. **HRMS (ESI) for C_73_H_108_ClN_18_O_32_P_2_^+^ [(M+H)^+^]/2** Calcd.:923.3307; Obs: 923.3282.

**Reference**

1. Dai, Z., Zhang, X. N., Nasertorabi, F., Cheng, Q., Li, J., Katz, B. B., Smbatyan, G., Pei, H., Louie, S. G., Lenz, H. J., Stevens, R. C., and Zhang, Y. (2020) Synthesis of site-specific antibody-drug conjugates by ADP-ribosyl cyclases. *Sci. Adv.* **6**, eaba6752
